# Chemical Characterization of Different Extracts of *Justicia secunda* Vahl and Determination of Their Anti-Oxidant, Anti-Enzymatic, Anti-Viral, and Cytotoxic Properties

**DOI:** 10.3390/antiox12020509

**Published:** 2023-02-17

**Authors:** Łukasz Świątek, Elwira Sieniawska, Kouadio Ibrahime Sinan, Gokhan Zengin, Anastazja Boguszewska, Benita Hryć, Kouadio Bene, Małgorzata Polz-Dacewicz, Stefano Dall’Acqua

**Affiliations:** 1 Department of Virology with SARS Laboratory, Medical University of Lublin, Chodzki 1, 20-093 Lublin, Poland; 2 Department of Natural Products Chemistry, Medical University of Lublin, Chodzki 1, 20-093 Lublin, Poland; 3 Department of Biology, Science Faculty, Selcuk University, Konya 42130, Turkey; 4 Medicofarma Biotech S.A., Zamenhofa 29, 20-453 Lublin, Poland; 5 Laboratoire de Botanique et Phytothérapie, Unité de Formation et de Recherche Sciences de la Nature, Université Nangui Abrogoua, Abidjan 02 BP 801, Côte d’Ivoire; 6 Department of Pharmaceutical and Pharmacological Sciences, University of Padova, Via Marzolo 5, 35131 Padova, Italy

**Keywords:** *Justicia*, phenolics, antiviral, bioactive agents, enzyme inhibitors

## Abstract

*Justicia secunda* Vahl. is a traditional medicinal plant in tropical regions, including West Africa. The present study examined the chemical profiles and biological properties of *J. secunda* extracts obtained with different solvents (dichloromethane, ethyl acetate, methanolic and aqueous: macerated and infused). Chemical components were characterized by liquid chromatography-mass spectrometry (LC-MS), and over 50 compounds were identified, including flavonoids, phenolic acids, and alkaloids. Antioxidant, enzyme inhibitory, cytotoxic, and antiviral properties were selected as biological properties. Total phenolic and flavonoid contents in methanol (58.07 mg gallic acid equivalent (GAE)/g and 13.07 mg rutin equivalent (RE)/g) and water (infused) (36.34 mg GAE/g and 8.52 mg RE/g) were higher than in other extracts. Consistent with the levels of total bioactive components, the methanol and water extracts exhibited stronger antioxidant abilities. However, the dichloromethane and ethyl acetate extracts were more active on α-amylase and α-glucosidase than other extracts. Aqueous extracts exerted selective anticancer properties toward human pharyngeal cancer cell lines, whereas the methanolic extract decreased the human herpesvirus type-1 (HHV-1) infectious titer by 2.16 log and the viral load by 1.21 log. Overall, *J. secunda* could be considered a multifunctional bioactive raw material in the preparation of potent applications to manage diseases related to oxidative stress, including cancer, diabetes, and Alzheimer’s.

## 1. Introduction

In the past ten years, the world population has been increasing daily, and the way of life of humans has undergone an intense change. Due to this fact, the prevalence of some diseases, including cancer [1], diabetes [2], or cardiovascular problems [3], is reaching a critical point worldwide, and we need to find global solutions. Synthetic drugs topped the list, but their long-term use raises serious concerns. In light of this, the importance of finding efficient drugs points out the opportunity to study natural products and plant species, which have attracted the interest of the scientific platform, especially, interest in un-investigated plants has been steadily increasing [4].

*Justicia secunda* Vahl. (eng. St. John’s bush) is a plant belonging to the Acanthaceae family. In Barbados, it is known as “Bloodroot” and in Venezuela as “Sanguinaria”, which refers to the red color of water observed when the plant is boiled. Traditional medicine of Barbadian locals describes the oral use of decoctions and infusions from the leaves of this plant to treat wound infections [5]. It is also used for bathing dogs suffering from skin rashes [6]. An ethnopharmacological survey carried out in Kikwit city (Kwilu province, southwest part of the Democratic Republic of the Congo) revealed that *J. secunda* leaves were used in traditional medicine to treat sickle cell disease. Importantly, further in vitro studies showed that *J. secunda* extract permits a change of shape of a sickle cell into a normal one with a maximal normalization rate (NRmax) of above 80% [7]. Moreover, the anthocyanin fraction isolated from *J. secunda* leaves extract also showed anti-sickling activity through direct binding with sickle cell deoxyhemoglobin molecules and stabilization of the sickle cell red blood cells’ membrane [8]. Traditional medicinal use of this plant to treat diabetes was also reported [9]. The anti-diabetic activity of this plant could be attributed to the presence of 2-caffeoyloxy-4-hydroxy-glutaric acid and the diastereomers secundarellone B and C, which were shown in vitro to inhibit α-glucosidase [9]. Interestingly, the literature data provide discrepant results concerning the antibacterial activity of *J. secunda*. Rojas et al. [10] reported that the aqueous extract exerted activity against *Escherichia coli* and ethanolic extract additionally against *Pseudomonas aeruginosa* and *Candida albicans* at concentrations of 25 μg/mL. The hexanoic extract inhibited only *Bacillus cereus*. Other Gram-positive bacteria, *Staphylococcus aureus* and β-hemolytic *Streptococcus*, were resistant to tested extracts [10]. Interestingly, Carrington et al. [5] reported that *Staphylococcus aureus* (ATCC 25923), *Pseudomonas aeruginosa* (ATCC 27853), and *Enterococcus feacalis* (clinical strain) were resistant to *J. secunda* methanolic and acetone extracts in concentrations range 1–200 mg/mL.

However, a comprehensive chemical and biological assessment of *J. secunda* still needs to be made available. With this in mind, we aimed to identify chemical components (by LC-MS technique) and to assess the biological activities (antioxidant, enzyme inhibitory, cytotoxic, and antiviral) of different extracts (dichloromethane, ethyl acetate, methanolic, aqueous (as macerated and infused)) of *J. secunda*. The obtained results could clarify the functional properties of *J. secunda* on the road from nature to pharmacy shelves.

## 2. Materials and Methods

### 2.1. Plant Materials and Extraction

The aerial parts of the plants (*Justicia secunda*) were collected from one locality (Nahio (Department of Issia, Haut-Sassandra region, Côte d’Ivoire) of Ivory Coast at the flowering period in the summer season of 2020. The plant was identified by a botanist (Dr. Kouadio Bene). Voucher specimens (KB-20-1016) were deposited at the herbarium at Selcuk University. The plant materials were thoroughly cleaned by washing with tap water and then rinsing with distilled water to remove soil and contaminants. The aerial parts were separated and dried in a well-ventilated, shaded environment at the room temperature. After 10 days of drying, the materials were ground into powder using a Retsch SM-200 laboratory mill and extracted within the same week. The powdered plant material was stored in a cool, dark, and well-ventilated area at around 20 °C. 

We used four solvents (dichloromethane, ethyl acetate, methanol, and water) to prepare plant extracts. The maceration method was chosen for the extracts, and 10 g of plant material was mixed with 200 mL of the solvents for 24 h at room temperature. The mixtures were then filtered with Whatman filter paper, and the solvents were removed with a rotary evaporator under reduced pressure at 40 °C. Traditional infusion was prepared using 10 g of plant material steeped in 200 mL of boiled water for 15 min. The mixture was subsequently filtered, frozen, and lyophilized for 48 h (−80 °C, under vacuum). All extracts were kept at 4 °C until analysis.

### 2.2. Determination of Total Phenolic, Flavonoid and Antioxidant and Enzyme Inhibitory Effects

Total phenolic content (TPC), total flavonoid content (TFC), DPPH radical scavenging, ABTS radical scavenging, cupric reducing antioxidant capacity (CUPRAC), ferric reducing antioxidant power (FRAP), metal chelating activity (MCA), phosphomolybdenum, inhibition of acetylcholinesterase (AChE), butyrylcholinesterase (BChE), tyrosinase, amylase, and glucosidase assays were performed as previously described [11,12]. Each sample was processed in triplicate.

### 2.3. LC-MS Analysis

Liquid chromatography—mass spectrometry analysis was performed using Agilent 1200 Infinity HPLC coupled to Agilent 6530B QTOF system (Agilent Technologies, Santa Clara, CA, USA). Chromatographic separation was performed on the C18 Gemini^®^ column (3 µm i.d., with TMS endcapping, 110 Å, 100 × 2 mm) supported with a guard column (Phenomenex Inc, Torrance, CA, USA). Solvent A (water with 0.1% formic acid *v*/*v*) and solvent B (acetonitrile with 0.1% formic acid) were pumped in the gradient: 0–60% B for 45 min., next 60–95% B for 1 min, and 95% B for 4 min, at the flow rate of 0.2 mL/min. A total of 10 μL of the sample was injected into the chromatographic column at 20 °C. MS detection was performed in positive and negative ion modes at a collision energies of 10 and 30 eV. Scanning was done in the range of 100–1000 *m*/*z*. The other conditions were as follows: drying gas temp: 275 °C, drying gas flow: 10 L/min, sheath gas temp: 325 °C, sheath gas flow: 12 L/min; nebulizer pressure: 35 psig, capillary V (+): 4000 V, skimmer 65 V, fragmentor 140 V. Compounds were tentatively identified based on their accurate masses and fragmentation patterns, also supported by the available literature sources.

### 2.4. Cytotoxicity Testing and Anticancer Selectivity

The cytotoxicity of *J. secunda* extracts was tested using a colorimetric microculture tetrazolium (MTT) assay according to the previously described methodology [13], and details were presented in Supplementary Materials. Briefly, the monolayers of selected cell lines in 96-well plates were incubated with serial dilutions of test extracts in cell media for 72 h. Afterwards, the MTT was performed to assess the cellular viability. Data analysis was conducted using GraphPad Prism, and the CC_50_ values (50% cytotoxic concentration) were evaluated. To evaluate the anticancer activity, the selectivity indexes (SI) were assessed in relation to VERO (SI = CC_50_VERO/CC_50_Cancer, SI > 1 indicates anticancer selectivity). For the FaDu cells, the influence of tested compounds on the cellular morphology was assessed during the 72 h incubation period using Olympus Provi CM20 Incubation Monitoring System (Evident Corporation, Hamburg, Germany).

### 2.5. Evaluation of the Anti-Herpesvirus Effect

The influence of *J. secunda* extracts on the Human Herpesvirus type-1 (HHV-1, aka. HSV-1) induced cytopathic effect (CPE) was assessed as previously described [13], and details are presented in the Supplementary Materials. For this purpose, the monolayers of VERO cells in 48-well plates were infected with 100-fold CCID_50_ (CCID_50_—50% cell culture infectious dose) of HHV-1 for 1 h. Afterwards, the monolayers were washed with PBS to remove unattached viral particles, tested compounds in non-toxic concentration were added, and incubation continued until the CPE was observed in the virus control (HHV-1 infected, untreated cells). Afterwards, the plates were observed using an inverted microscope (CKX41, Olympus Corporation, Tokyo, Japan) for possible inhibition of CPE by tested extracts, and the results were recorded. The plates were thrice frozen (−72 °C) and thawed; the samples were collected and stored at −72 °C until used in the end-point virus titration assay and DNA isolation. Acyclovir was used as a reference antiviral drug.

Samples collected from antiviral assays were subjected to an end-point dilution assay to evaluate the HHV-1 titers. The difference (Δlog) of HHV-1 infectious titer (logCCID_50_/mL) in the samples treated with *J. secunda* extracts and in the virus control (VC) from the same experiment (Δlog = logCCID_50_VC − logCCID_50_JS) were calculated. Data analysis was conducted using GraphPad Prism.

The DNA was isolated using a commercially available kit (QIAamp DNA Mini Kit, Cat#51304, QIAGEN GmbH, Hilden, Germany) following the manufacturer’s instructions. The real-time PCR amplification was performed using SybrAdvantage qPCR Premix (Takara Bio Inc., Kusatsu, Shiga, Japan) and primers (UL54F-5′ CGCCAAGAAAATTTCATCGAG 3′, UL54R-5′ ACATCTTGCACCACGCCAG 3′) on the CFX96 real-time thermal cycler (Bio-Rad Laboratories, Inc., Pleasanton, CA, USA). The HHV-1 viral load in the tested samples was assessed in relation to virus control based on the relative quantity (ΔCq) method using CFX Manager™ Dx Software (Bio-Rad Laboratories, Hercules, CA, USA).

### 2.6. Data Analysis

Data were presented as mean ± standard deviation. One-way analysis of variance with Tukey’s post-hoc test was achieved; *p* < 0.05 was considered statistically significant. Then Pearson correlation coefficient between the chemical compounds and the antioxidant and enzyme inhibitory activity were calculated. Pearson’s coefficient higher than 0.7 was considered statistically significant. All analysis were done under R v 4.1.2 software.

## 3. Results and Discussion

### 3.1. Total Phenolic and Flavonoid Contents

**Figure 1:**
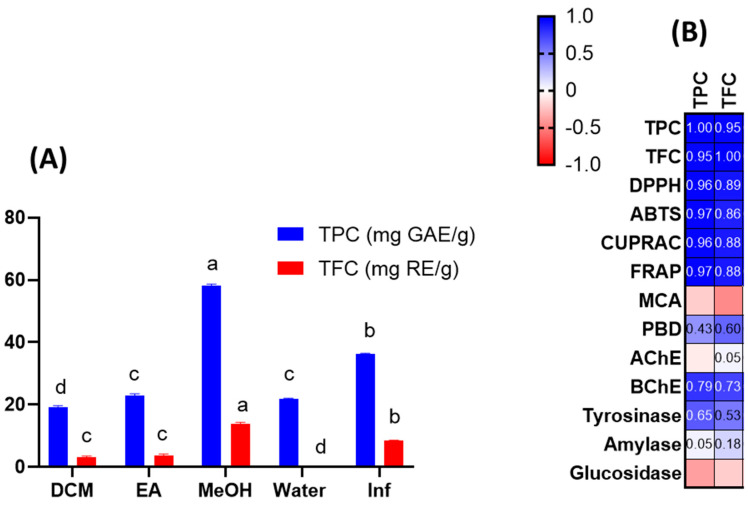
Total phenolic (TPC) and total flavonoid (TFC) content of the tested extracts (**A**). Pearson’s correlation values between total bioactive compounds and antioxidant and enzyme assays (**B**). Values are reported as mean ± SD of three parallel measurements. GAE: gallic acid equivalents; RE: rutin equivalents. Different letters (a–d) in the columns indicate significant differences in the tested extracts (“a” indicated the highest content, *p* < 0.05). ABTS, 2,2′-azino-bis (3-ethylbenzothiazoline) 6-sulfonic acid; AChE, acetylcholinesterase; BChE, butyrylcholinesterase; CUPRAC, cupric ion reducing antioxidant capacity; DPPH, 1,1-diphenyl-2-picrylhydrazyl; FRAP, ferric ion reducing antioxidant power; MCA, metal chelating activity; PBD, phosphomolybdenum activity; AChE: acetylcholinesterase; BChE: butrylcholinesterase.

Extracts were tested for their total phenolic and flavonoid content using spectrophotometric techniques. As can be seen from Figure 1A, the highest concentration of total bioactive compounds was detected in methanolic extract (TPC: 58.07 mg gallic acid equivalent (GAE)/g: TFC: 13.70 mg rutin equivalent (RE)/g), followed by infusion (TPC: 36.34 mg GAE/g: TFC: 8.52 mg RE/g) and ethyl acetate extract (TPC: 22.98 mg GAE/g: TFC: 3.65 mg RE/g). The total amount of bioactive compounds was lowest in the dichloromethane and aqueous extracts. Total bioactive compound amounts were clearly influenced by the solvents used. Based on our results, methanol and water (as an infusion) could be effective solvents for further applications of *J. secunda*. Few publications in the literature have reported the total phenolic content of *J. secunda* extracts or fractions [14,15,16]. In the reports, the authors used different standards to explain the total content of bioactive compounds in dry or fresh samples, therefore our results could not be compared with most previous reports. In a study conducted by John et al. [16], the total phenolic and flavonoid contents were reported for six *Justicia* species and the highest level was reported for the leaf extract of *J. adhatoda* with 38.75 mg GAE/100 g extract, which was lower than those of our tested extracts. Moreover, the total phenolic and flavonoid contents were reported as 1.33–5.01 mg GAE/100 g and 0.18–1.30 mg catechin equivalent (CE)/100 g for *J. spicigera* extracts, respectively in another study [17]. Naqvi et al. [18] reported the total phenolic level as 30.4–80.9 mg GAE/100 g extract in *J. camara* and *J. adhatoda* extracts. Based on the results of earlier reports, we concluded that *J. secunda* could be considered a valuable source of phenolic compounds.

### 3.2. Chemical Characterization

Applying high-performance liquid chromatography combined with electrospray ionization-quadrupole time-of-flight mass spectrometry enabled the tentative identification of over numerous compounds in various extracts obtained from *J. secunda* leaves (Table 1). Spectrometric data collected in negative and positive ion mode for all compounds are presented in Supplementary Table S1.

The first group of compounds identified in *J. secunda* leaves were flavonoids. They were found in infusion and methanolic extract and also in fewer number in ethyl acetate extract. Among flavonoids, mainly glycosidic derivatives of tetrahydroxyflavone, trihydroxyflavone, trihydroxymethoxyflavone, and tetrahydroxyflavonol were observed and tentatively identified as luteolin, apigenin, diosmetin, and quercetin, respectively. Di- and trisaccharidic moieties substituted on flavonoids comprised glucose, rhamnose, apiose, xylose, and acetylpentose (tentatively identified as acetylapiose). Phenolic acids substituted on flavonoids were also observed. Two compounds: luteolin 7-O-[β-glucopyranosyl-(1→2)-β-rhamnosyl-(1→6)] β-glucopyranoside and luteolin 7-O-[β-apiofuranosyl-(1→2)]-β-xylopyranoside were previously identified in *J. secunda* leaves by Koffi et al. [14] by nuclear magnetic resonance (^1^H NMR,^13^C NMR). Moreover, Koffi et al. [15] identified luteolin 7-O-rutinoside by HPLC-ESI-MS/IonTrap in the same plant species. The abovementioned compounds were identified in our extracts.

Polyphenols were also represented by phenolic acids. Benzoic acid derivatives were present in infusion, aqueous and methanolic extracts. Glycosides of salicylic, syringic, dihydroxy- and trihydroxybenzoic (gallic) acids were tentatively identified. Hydroxycinnamic and caffeic acid derivatives were found in methanolic extract and infusion. Moreover, non-phenolic carboxylic acids derivatives were present in infusion, aqueous, and methanolic extracts.

According to Corrêa and Alcântara [19], lignans are one of the chemical classes found in the different species of the genus *Justicia*. We found four isomers characterized by the same precursor ion [M-H]^-^ at *m*/*z* 355.0612 (predicted) but with different retention times, which were tentatively identified as lignan derivatives, probably 10-(1,3-benzodioxol-5-yl)-5H-benzo[c]furo[3,2-g]chromen-5-one isomers. They were present only in the infusion. Koffi et al. [15] also found precursor ion at *m*/*z* 355 ([M-H]^−^), which they identified as justiflorinol with molecular formula C_19_H_16_O_7_. Nevertheless, the molecular formula calculated for the precursor ion [M-H]^−^ at *m*/*z* 355.0612 (C_22_H_12_O_5_) found in our extract varied and was identified as a different compound. One lignan derivative ([M-H]^−^ at *m*/*z* 505.2079, predicted) was found in the methanolic extract.

Moreover, cyclohexanone derivatives, mainly glucosides, were found in all obtained extracts except the aqueous one. According to data obtained for *J. aequilabris* [20], a compound characterized by the precursor ion [M+COOH]^−^ at *m*/*z* 431.1923 (predicted) was identified as roseoside. Two different cyclohexanone derivatives were tentatively identified as dihydroroseoside and 9-hydroxy-7-megastigmen-3-one glucoside. Furthermore, decalin glucoside derivatives tentatively identified as ophiogonoside A isomers were present in all extracts except the aqueous one.

Alkaloids are one of the compound classes found in the species belonging to the genus *Justicia* [19,21]. NMR analyses performed by Theiler et al. [22] revealed the presence of secundarellone B/C (racemate) and secundarellone A in the leaves of *J. secunda*. These compounds can be classified as pyrrolidone alkaloids. Secundarellone B/C and one isomer of secundarellone A were found in dichloromethane extract, and the second one of the isomers of secundarellone A was identified in all obtained extracts.

### 3.3. Antioxidant Capacity

Antioxidant has been one of the most used terms in pharmaceutical, nutraceutical, and cosmetic applications over the past decade. Antioxidant compounds could be considered powerful protective agents against the attacks of free radicals, which are the main trigger for developing severe health problems such as cancer, diabetes, or cardiovascular diseases. In this sense, discovering new sources of antioxidants is crucial to treating the mentioned diseases. In the chemical industry, some compounds, including butylated hydroxyanisole (BHA), butylated hydroxytoluene (BHT), and propyl gallate (PG), have been chemically produced, but their use raises public concerns such as hepatotoxicity or cancerogenic influence on the human organism [23]. Therefore, synthetic antioxidants should be swapped with natural alternatives, and plants serve as a rich source of such antioxidants.

Given the preceding information, we examined the antioxidant capacities of *J. secunda* extracts using various chemical assays, such as free radical quenching, reducing power, and metal chelation. The results are presented in Table 2. The DPPH and ABTS assays are the most prevalent antioxidant assays and provide information on plant extracts’ radical-scavenging capacity. In both assays, the tested methanolic and infused extracts were the most active, while the weakest ability was exerted by the dichloromethane extract. In addition, the macerated and infused aqueous extracts showed different abilities in the free radical scavenging assays. In general, the antioxidant mechanism is based on hydrogen or electron-donation abilities. Reducing power assays have been utilized to assess the electron-donation capacity of a compound or extract. CUPRAC and FRAP are the two most important reducing power assays based on the transformation of Cu^2+^ to Cu^+^ and Fe^3+^ to Fe^2+^, respectively. Table 2 shows that similar to radical scavenging assays, methanolic and infusion extracts were found to possess the most potent reducing abilities. In addition, the extracts contained higher amounts of the total phenolic and flavonoid when compared to other extracts. Both radical scavenging (R^2^ was 0.96 for DPPH and TPC; was 0.97 for ABTS and TPC) and reducing power assays (R^2^ was 0.96 for CUPRAC and TPC; was 0.97 for FRAP and TPC) correlated strongly with the total bioactive content at this point (Figure 1B). Consistent with our approach, several researchers reported a linear correlation between the levels of total bioactive compounds and these parameters [24,25,26]. In addition to the positive correlation between total bioactive compounds and free radical scavenging/reduction assays, the presence of some compounds can be attributed to the observed significant antioxidant properties of methanol and infused extracts. For example, rutin was only detected in the tested methanol extract and it has already been shown to be a powerful antioxidant [27]. Besides rutin, caffeic acid derivatives was only found in the methanol extract and some researchers have reported antioxidant properties of caffeic acid [28,29]. Regarding infused extract, particularly the presence of apigenin and hydroxycinnamic acid were only detected in infused extract and these compounds may contribute to the observed antioxidant properties [30,31]. In the literature, several researchers reported significant free radical scavenging and reducing abilities of members of the *Justicia* genus. For example, Koffi et al. [14] examined *J. secunda* leaves extracts for ABTS radical scavenging, and the ability was reported as 218 µmol TE/g for non-concentrated extract. In another study by Anyasor et al. [32], the hot-water extract of *J. secunda* leaves showed a stronger ability to scavenge free radicals and reduce potency than the cold-water extract, consistent with our presented results. Significant free radical scavenging and reducing potential of the methanolic extract (80%) of *J. secunda* leaves was reported in a previous study by Onoja et al. [33]. Phosphomolybdenum assays involve the transformation of Mo (VI) to Mo (V) under acidic conditions by antioxidant compounds. In contrast to other reducing power assays, the best ability was recorded in the ethyl acetate extract with 1.66 mmol TE/g, followed by methanol and dichloromethane. This case could be explained by the presence of non-phenolic antioxidants such as ascorbic acid and tocopherols. Thus, the correlation between total phenolic and phosphomolybdenum was weak (R^2^: 0.43). Several researchers who reported a weak correlation between total phenolic and phophomolybdenum assay concurred with our findings [34,35]. Transition metals play a role in the Fenton reaction, which leads to the formation of hydroxyl radicals. As a result, when transition metals are chelated, the production of this radical is inhibited. Therefore, metal chelation is considered to be an important antioxidant mechanism. In contrast to other assays, the highest metal chelating ability was provided by the aqueous extract with 35.91 mg EDTAE/g, followed by ethyl acetate and methanolic extracts. Surprisingly, the infusion showed no ability to metal chelating. The presence of non-phenolic chelating agents, such as peptides and polysaccharides in the tested extracts, could explain the conflicting results. Overall, we provided a comprehensive antioxidant profile of different extracts from *J. secunda*. The methanolic and infused extracts of *J. secunda* exhibited greater antioxidant activity than other extracts, and this finding could be used to design functional applications utilizing *J. secunda* in future research.

### 3.4. Enzyme Inhibitory Properties

Enzymes play crucial roles in developing effective therapeutic strategies to combat global health issues. In this sense, the majority of drugs have been designed to inhibit specific enzymes. This phenomenon is known as the enzyme inhibition theory [36]. For example, the main problem in diabetics is high blood glucose levels, and this could be managed by inhibiting amylase and glucosidase, the main starch-degrading enzymes [37]. Similar correlations could be made between the inhibition of acetylcholinesterase and Alzheimer’s patients, who have lower acetylcholine levels compared to healthy individuals [38]. With this in mind, some compounds have been synthesized through chemical synthesis, but they have undesirable side effects, such as gastrointestinal disturbances and toxicity [39,40,41]. Consequently, more secure, and efficient phytochemical-based inhibitors are desired.

In light of the above information, we examined the enzyme-inhibitory properties of *J. secunda* extracts against AChE, BChE, tyrosinase, amylase, and glucosidase. The results are presented in Table 3. The best AChE inhibitory ability was observed by the ethyl acetate extract with 1.63 mg GALAE, but it was not statistically different from dichloromethane (1.51 mg GALAE/g) (*p* > 0.05). Regarding the BChE inhibitory abilities, only two extracts (methanolic and ethyl acetate) were active on the enzyme, and the methanolic extract was more active than the ethyl acetate one. In both AChE and BChE assays, macerated and infused aqueous extracts were not active. The observed anti-cholinesterase ability of the methanolic extract might be explained by the presence of rutin, which was only detected in the methanolic extract. Similar to our approach, the anti-cholinesterase ability of rutin and its mechanism have been reported in several studies [42,43]. In addition to rutin, the observed ability to inhibit cholinesterase can be attributed to caffeic acid derivatives. In earlier studies [44,45], caffeic acid has been reported as a significant anti-Alzheimer’s agent. There is a dearth of data in the scientific literature about the cholinesterase-inhibiting effects of the *Justicia* genus. In contrast to our results, Rawa et al. [46] reported that the methanolic extract of *J. gendarussa* leaves was not active on both AChE and BChE. Previous research by Gupta and Gupta [47] found that both the leaf and stem of *J. gendarussa* showed weak AChE inhibitory properties.

Similar to the cholinesterase inhibitory properties, the aqueous extracts exhibited lower inhibitory effects on amylase and glucosidase compared to other extracts. The ethyl acetate extract was the most active on the anti-diabetic enzymes, but its activity was not statistically different from dichloromethane extract. The observed inhibitory effects on amylase and glucosidase did not correlate with the total bioactive compounds. As can be seen from Table 2, diosmetin was only detected in ethyl acetate extract and its presence can be attributed to the observed antidiabetic ability of the extract. In an earlier study by Yuan et al. [48], diosmetin as well as luteolin and quercetin exhibited a significant amylase inhibitory effect. The compounds in the methanol extracts such as rutin and caffeic acid derivatives can also contributed to the observed antidiabetic ability [49,50]. The amylase inhibitory effect of the aqueous extract of *J. carnea* was reported to be moderate [51], which is similar to our findings. In another study, Ani et al. [52] studied the anti-amylase and anti-glucosidase abilities of different extracts (ethanolic, methanolic, and aqueous) of *J. carnea* leaves and found that the ethanolic extract was the most active. In contrast to our results, the methanolic extract of *J. adhatoda* leaves showed low amylase inhibitory activity with an inhibitory value of less than 50% [53].

Melanogenesis is an essential process that shields humans from the sun’s ultraviolet rays. Tyrosinase is a crucial enzyme in melanogenesis reactions, and inhibiting it could alleviate hyperpigmentation problems [41]. As can be seen from the Table 3, the tested extracts can be ranked according to decrease tyrosinase inhibitory activity as follows: methanolic > aqueous > infusion > dichloromethane > ethyl acetate. As can be seen from Figure 1, the observed tyrosinase inhibitory ability correlated moderately with the total phenolic and flavonoid content of the tested extracts (R^2^ < 0.7). Based on the identified compounds from Table 1, the observed anti-tyrosinase ability can be attributed to some compounds. For example, several compounds, including rutin, luteolin and caffeic acid, have already been reported as effective anti-tyrosinase agents [54,55]. In a recent study by Basit et al. [56], the tyrosinase inhibitory effect of *n*-butanol extract of *J. vahlii* leaves was reported as 193.21 mg KAE/g, which was higher than our results. The anti-tyrosinase inhibitory effect of the ethanolic extract (50%) of *J. adhatoda* leaves was found to be less than 50% in a previous study by Ito et al. [57]. The current study is, to our knowledge, the first to describe the enzyme-inhibiting properties of *J. secunda*. The results could shed light on the potential applications of *J. secunda* in nutraceutical, cosmetic, and pharmaceutical practices.

### 3.5. Cytotoxicity Evaluation and Anticancer Selectivity

**Figure 2:**
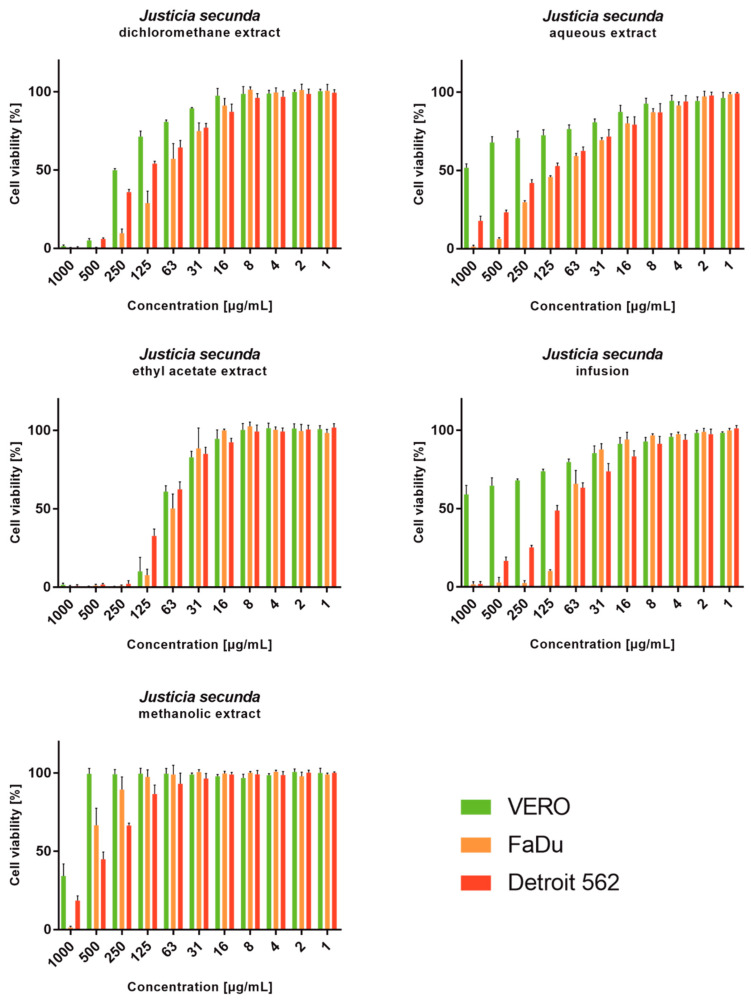
Viability of cell lines treated with different concentrations of *Justicia secunda* extracts (VERO—cell line from the kidney of an African green monkey; FaDu—cell line from hypopharyngeal carcinoma; Detroit 562—cell line from pharyngeal carcinoma).

**Figure 3:**
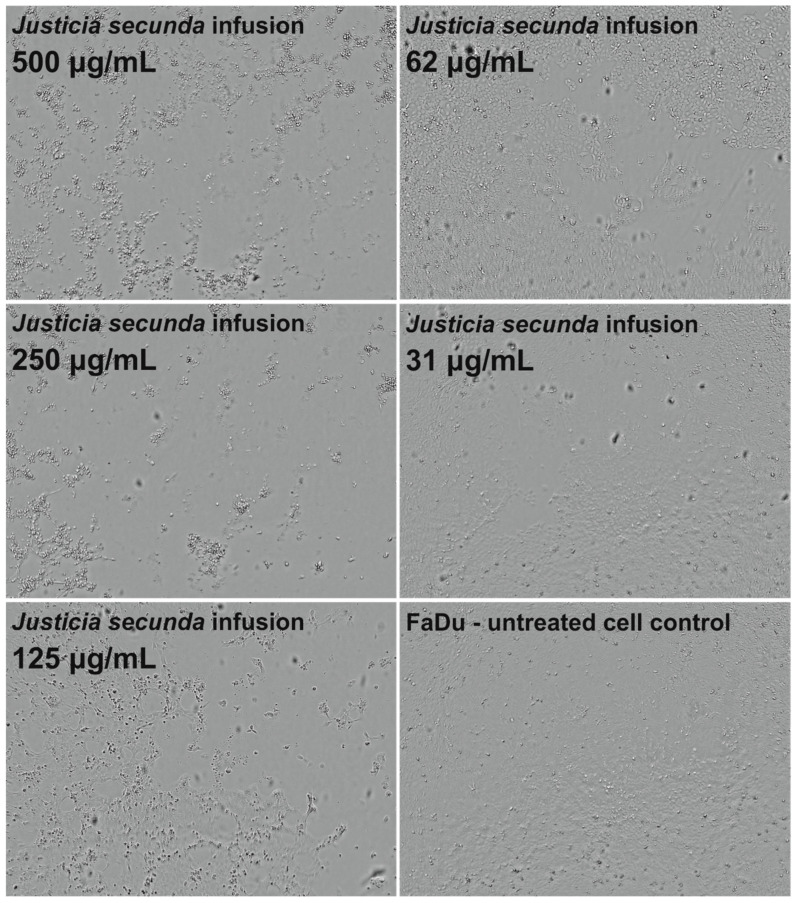
Influence of *Justicia secunda* infusion on the morphology of FaDu cells’ monolayer after 72 h incubation.

*J. secunda* aqueous extract and infusion showed the lowest cytotoxicity toward normal VERO cells, and the exact CC_50_ values could not be evaluated since they were above the tested concentration range (Figure 2). Based on the classification of plant extract’s cytotoxicity, the methanolic and aqueous extracts, as well as the infusion, can be labelled as non-toxic to normal cells [58]. The highest toxicity toward all cell types was observed for the ethyl acetate extract with CC_50_ between 61.18 and 79.57 μg/mL (Table 4). Despite SI of 1.11 calculated for ethyl acetate extract on FaDu, it was not found to be significant (*p* > 0.5). *J. secunda* dichloromethane extract showed substantial anticancer activity (*p* < 0.01) toward FaDu and Detroit with SI of 2.83 and 1.75, respectively. Notably, also methanolic extract exerted anticancer selectivity, but the observed CC_50_ values point to low toxicity. The highest anticancer potential and selectivity toward both pharyngeal carcinomas were found for *J. secunda* aqueous extract and infusion. The influence of infusion in the 500, 250, 125, 62 and 31 μg/mL concentrations on the morphology of FaDu cells monolayer is presented in Figure 3. At 500 and 250 μg/mL concentrations, the cellular monolayer was utterly destroyed after 72 h of incubation, and no FaDu cells could be noticed. The 125 μg/mL concentration also induced damage to the monolayer, but some intact cells were visible. The cellular monolayer was observed in the lower concentrations, 62 and 31 μg/mL, but it was noticeably less dense than in the cell control. Olympus Provi CM20 allowed for constant monitoring of cellular monolayer and observation of the changes induced by different concentrations of tested extracts. It was found that most of the damage to the FaDu monolayer induced by the INF 500 μg/mL occurred during 24 h of incubation. The infusion at 250 μg/mL showed a similar cytotoxic effect after 44 h of incubation.

Onochie et al. [59] reported that *J. secunda* leaves ethanolic extract caused increased lipid profile values, creatinine and blood urea levels in Wistar rats, which may indicate potential negative cardiac and renal influence. Moreover, the calculated LD_50_ (50% lethal dose) of the extract was determined to be 3800 mg/kg body weight, which also raises potential safety concerns [59]. However, these effects were only described for ethanolic extract, and it must be noted that, in most cases, traditional medicine uses *J. secunda* infusions or decoctions, which may exert different safety profiles. Nevertheless, our in vitro experiments also indicate lower toxicity of aqueous extract and infusion to normal cells than the extracts obtained using organic solvents, especially dichloromethane and ethyl acetate. It should be emphasized that this report is the first one depicting the cytotoxicity and anticancer potential of extracts obtained from the aerial parts of *J. secunda*.

### 3.6. Antiviral Activity

**Figure 4:**
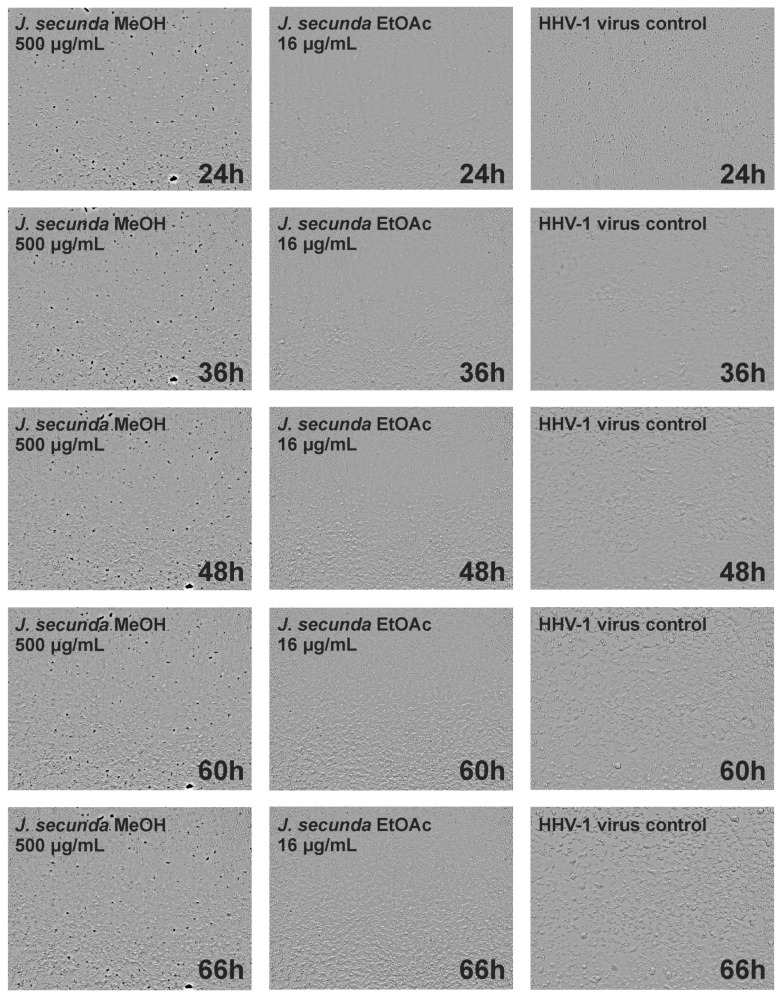
Real-time microscopic monitoring of the antiviral assay.

Incubation of *J. secunda* methanolic extract (500 μg/mL) with HHV-1 infected VERO cells decreased the intensity of virus-induced cytopathic effect (CPE). However, there were still noticeable signs of CPE present. The rest of the tested extracts did not influence the formation of CPE. The formation of CPE was observed every 2 h during a 72 h incubation period using Olympus Provi CM20. Live-cell imaging allowed for real-time monitoring of morphological changes induced by the HHV-1 infection in the VERO cells’ monolayer. A comparison between the development of CPE in virus-infected cells treated with *J. secunda* methanolic extract (500 μg/mL) or *J. secunda* ethyl acetate extract (16 μg/mL), and the CPE in the virus control (VC) is presented in Figure 4. After 36 h of incubation, the CPE was clearly visible in the VC. However, in the cells treated with *J. secunda* ethyl acetate extract, only early signs of CPE, including cell rounding and several areas of focal degeneration, were visible, and *J. secunda* methanolic extract protected cells from virus-induced damage. After the 48 h incubation period, the VC showed profound CPE, with ballooning degeneration, vacuolization, and polykaryon (syncytia) formation. Moreover, the HHV-1-infected cells treated with *J. secunda* ethyl acetate extract showed significant CPE, and occasional cells treated with *J. secunda* methanolic extract showed early signs of CPE (cell swelling, rounding, and clumping). Continued incubation (Figure 4; 60 h and 66 h) resulted in the complete destruction of the cellular monolayer in VC and infected cells treated with *J. secunda* ethyl acetate extract. Despite signs of CPE, *J. secunda* methanolic extract after 66 h exerted a noticeable decrease in virus-induced damage compared to VC. Further incubation, up to 72 h, did not induce significant changes in the observed cells. Acyclovir (60 μg/mL), a reference antiviral drug, inhibited the development of the CPE.

**Figure 5:**
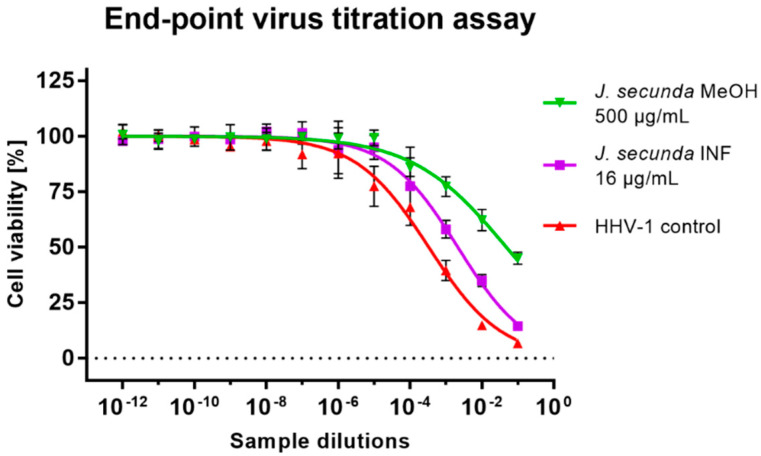
The end-point titration of HHV-1 infectious titer in the samples treated with *Justicia secunda* methanolic extract or infusion in relation to the virus control.

**Figure 6:**
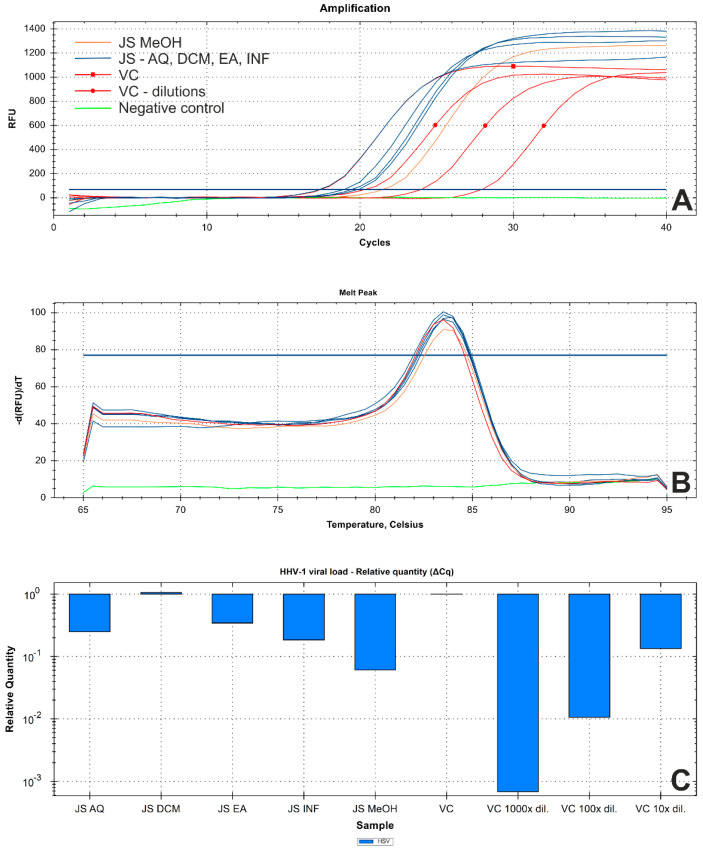
Results of real−time PCR analysis of the HHV−1 viral load in tested samples ((**A**)—amplification curve; (**B**)—DNA melt peak; (**C**)—relative quantitation using ΔCq method; Abbreviations: JS—DNA isolates from *Justicia secunda*-treated HHV−1-infected cells; VC—DNA isolate from virus control, and series of tenfold dilutions; Extracts: DCM—dichloromethane; EA—ethyl acetate; MeOH—methanol; AQ—water; INF—infusion).

Subsequent virus titration showed that *J. secunda* methanolic extract (500 μg/mL) managed to decrease the HHV-1 infectious titer by 2.16 ± 0.25 log (Figure 5). Other tested extracts showed inhibition below 1 log. However, significant antiviral activity can only be reported for samples decreasing the infectious titer by at least 3 log. End-point virus titration showed that acyclovir (60 μg/mL) successfully blocked the HHV-1 replication and viral infectivity was not detected. To further evaluate the influence of *J. secunda* on the replication of HHV-1, the viral load was measured using the real-time PCR technique (Figure 6A). The HHV-1 viral load in the tested samples was assessed in relation to virus control based on the relative quantity (ΔCq) method (Figure 6C). It was found that *J. secunda* methanolic extract (500 μg/mL) inhibited the replication of HHV-1, decreasing the viral load by 1.21 log compared to the virus control. Virus-infected cells treated with *J. secunda* dichloromethane extract showed a titer of HHV-1 comparable to the virus control. In contrast, ethyl acetate, aqueous, and infusion extracts reduced the viral load by 0.46, 0.6, and 0.73 log, respectively. The viral DNA was not detected in samples obtained from HHV-1-infected cells treated (60 μg/mL) with acyclovir. The melt curve analysis showed a single melting peak at 83.5–84 °C (Figure 6B), confirming the presence of the same amplicon in all tested samples, except negative control (uninfected cells). Considering the presented data, it can be concluded that *J. secunda* extracts failed to exert a significant antiviral effect against HHV-1. However, herein the anti-herpesviral activity of extracts from the aerial parts of *J. secunda* has been evaluated for the first time. It was previously reported that *J. procumbens* var. *leucantha* has substantial inhibitory activity against vesicular stomatitis virus (VSV), and lignans and their glycosides (e.g., justicidins A and B, diphyllin, diphyllin apioside and diphyllin apioside-5-acetate) were found to be responsible for the observed effect [60]. Lignans from *Justicia* spp. also demonstrated antiviral activity against HIV-1 [61,62,63]. Arylnaphthalene lignans from *J. procumbens*, justatropmers A–H, showed potent anti-HIV-1 activity [63]. The anti-HIV-1 activity was also reported for 70%-fractionated ethanolic extract of *J. gendarussa* on HIV-infected MOLT-4 cells using syncytia formation assay and HIV p24 antigen assay [64]. Interestingly, anisotine, an alkaloid from *J. adhatoda*, was shown in silico to inhibit the main protease (Mpro) of the SARS-CoV-2 [65]. We have also found lignan derivatives in the *J. secunda* extracts, which may be related to the observed inhibition of HHV-1-induced CPE formation and reduction of infectious titer and viral load by the methanolic extract, since lignans were also reported to inhibit herpesviruses [61]. However, the concentration of lignans in *J. secunda* extracts might have been insufficient for higher inhibition of HHV-1 replication. That is why it could be beneficial to perform a more detailed analysis of *J. secunda*, including the isolation of bioactive fractions and pure compounds responsible for the observed bioactivity.

## 4. Conclusions

In the current study, we focused on the chemical profile and biological activities of extracts of *J. secunda* obtained with different solvents. Both chemical profiles and biological abilities were influenced by the extraction solvent. In general, the best antioxidant activity was observed for the methanolic and aqueous extracts. However, different results were obtained considering each enzyme inhibitory assay. LC-MS analysis revealed the presence of biologically active compounds, including apigenin glucosides (infusion and methanolic extract), rutin (methanolic extract), luteolin and its glucosides (infusion, ethyl acetate and methanolic extracts), and alkaloids, namely secundarellones B/C in the dichloromethane extracts and isomers of secundarellone A in all tested extracts. Lignan derivatives were found in the infusion and methanolic extract. The highest toxicity toward all cell types was observed for the ethyl acetate extract. Whereas the highest anticancer potential and selectivity toward both pharyngeal carcinomas, FaDu and Detroit 562, were found for aqueous extract and infusion. Our results show that *J. secunda* has significant biological properties, making it a promising candidate as a coadjutant agent for developing therapeutics, dietary supplements, and cosmetics. However, further studies are strongly suggested to isolate active compounds responsible for biological activities

## Figures and Tables

**Table 1:** The secondary metabolites determined in *J. secunda* extracts.

No.	Molecular Formula	Identification	Extracts
1.	C_5_H_10_O_6_	Carboxylic acid derivative	MeOH, H_2_O, INF
2.	C_5_H_8_O_6_	Carboxylic acid derivative	H_2_O, INF
3.	C_6_H_10_O_8_	Unknown	MeOH, INF
4.	C_12_H_15_NO_6_	Secundarellone B/C (racemate)	DCM
5.	C_6_H_8_O_7_	Citric acid	MeOH, INF
6.	C_12_H_15_NO_5_	Secundarellone A isomer 1	DCM
7.	C_12_H_15_NO_5_	Secundarellone A isomer 2	DCM, EA, MeOH, H_2_O, INF
8.	C_10_H_11_NO_6_	Unknown	INF
9.	C_22_H_12_O_5_	Lignan derivative	INF
10.	C_15_H_20_O_10_	Syringic acid glucoside	H_2_O, INF
11.	C_7_H_12_O_5_	Carboxylic acid derivative	H_2_O, INF
12.	C_13_H_16_O_8_	Salicylic acid glucoside	MeOH, INF
13.	C_13_H_16_O_9_	Dihydroxybenzoic acid *O*-glucoside (2,4-dihydroxybenzoic acid *O*-glucoside)	INF
14.	C_12_H_14_O_8_	Dihydroxybenzoic acid *O*-pentoside	MeOH, H_2_O, INF
15.	C_22_H_12_O_5_	Lignan derivative (10-(1,3-benzodioxol-5-yl)-5H-benzo[c]furo [3,2-g]chromen-5-one isomer)	INF
16.	C_13_H_16_O_9_	Unknown	MeOH, H_2_O, INF
17.	C_22_H_12_O_5_	Lignan derivative (10-(1,3-benzodioxol-5-yl)-5H-benzo[c]furo [3,2-g]chromen-5-one isomer)	INF
18.	C_15_H_18_O_9_	Caffeoyl glucoside	MeOH, INF
19.	C_18_H_28_O_9_	Hydroxyjasmonic acid glucoside	MeOH, INF
20.	C_19_H_30_O_8_	Roseoside	DCM, EA, MeOH, INF
21.	C_20_H_26_O_12_	Hydroxycinnamic acid *O*-pentoside-glucoside	MeOH, INF
22.	C_22_H_12_O_5_	Lignan derivative (10-(1,3-benzodioxol-5-yl)-5H-benzo[c]furo [3,2-g]chromen-5-one isomer)	INF
23.	C_17_H_22_O_13_	Trihydroxybenzoic acid *O*-dipentoside (Gallic acid *O*-dipentoside)	INF
24.	C_12_H_14_O_9_	Trihydroxybenzoic acid *O*-pentoside (Gallic acid *O*-pentoside)	MeOH, INF
25.	C_19_H_32_O_8_	Cyclohexanone derivative glucoside (Dihydroroseoside)	DCM, EA, MeOH, INF
26.	C_14_H_14_O_8_	Caffeic acid derivative	MeOH
27.	C_9_H_8_O_3_	Hydroxycinnamic acid	INF
28.	C_11_H_16_O_3_	Unknown	DCM, EA, MeOH, INF
29.	C_33_H_40_O_20_	Luteolin 7-*O*-[β-glucopyranosyl-(1→2)-β-rhamnosyl-(1→6)] β-glucopyranoside	EA, MeOH, INF
30.	C_33_H_40_O_19_	Trihydroxyflavone di-*O*-hexoside-*O*-rhamnoside (apigenin 7-*O*-glucoside-glucoside-rhamnoside)	MeOH, INF
31.	C_21_H_36_O_8_	Decalin derivative	DCM, EA, MeOH, INF
32.	C_34_H_42_O_20_	Trihydroxymethoxyflavone di-*O*-hexoside-*O*-rhamnoside (diosmetin 7-*O*-glucoside-rhamnoside-glucoside)	MeOH, INF
33.	C_27_H_30_O_15_	Tetrahydroxyflavone *O*-hexoside-*O*-rhamnoside (luteolin-7-*O*-rutinoside)	EA, MeOH, INF
34.	C_27_H_30_O_16_	Rutin	MeOH
35.	C_19_H_32_O_7_	Cyclohexanone derivative glucoside (9-Hydroxy-7-megastigmen-3-one glucoside)	DCM, EA, MeOH, INF
36.	C_26_H_34_O_10_	Lignan derivative	MeOH
37.	C_19_H_28_O_7_	Cyclohexanone derivative	DCM, EA, MeOH
38.	C_37_H_38_O_19_	Tetrahydroxyflavone *O*-hexoside-*O*-rhamnoside derivative (luteolin *O*-hydroxyferuloyl -*O*-rutinoside)	INF
39.	C_21_H_38_O_8_	Decalin glucoside derivative (Ophiopogonoside A isomer)	EA, MeOH, INF
40.	C_26_H_28_O_15_	Tetrahydroxyflavone *O*-pentoside-hexoside; (graveobioside A = luteolin 7-*O*-(2-apiosyl)glucoside)	EA, MeOH, INF
41.	C_13_H_15_NO_3_	Unknown	DCM
42.	C_25_H_26_O_14_	Luteolin 7-*O*-[β-apiofuranosyl-(1→2)]-β-xylopyranoside	EA, MeOH, INF
43.	C_19_H_34_O_10_	Cyclohexanone derivative glucoside	DCM, EA, MeOH, INF
44.	C_26_H_28_O_14_	Tetrahydroxyflavone *O*-pentoside-*O*-rhamnoside (luteolin *O*-pentoside-*O*-rhamnoside)	EA, MeOH, INF
45.	C_17_H_30_O_7_	Unknown	DCM, EA, MeOH, INF
46.	C_21_H_38_O_8_	Decalin glucoside derivative (Ophiopogonoside A isomer)	DCM, EA, MeOH, INF
47.	C_19_H_36_O_10_	Rhodiooctanoside	DCM, EA, MeOH,
48.	C_27_H_28_O_15_	Tetrahydroxyflavone *O*-pentoside-*O*-acetylpentoside (luteolin *O*- apiofuranosyl -*O*-acetylapiofuranosyl)	MeOH
49.	C_25_H_26_O_13_	Trihydroxyflavone di-*O*-pentoside (apigenin di-*O*-pentoside)	MeOH, INF
50.	C_26_H_28_O_14_	Trihydroxymethoxyflavone di-*O*-pentoside (diosmetin 7-*O*-apiofuranosyl-xylopyranoside)	EA, MeOH, INF
51.	C_27_H_30_O_14_	Trihydroxymethoxyflavone *O*-pentoside *O*-rhamnoside (diosmetin *O*-apiofuranosyl- *O*-rhamnoside)	EA, MeOH, H_2_O, INF
52.	C_28_H_30_O_15_	Trihydroxymethoxyflavone *O*-pentoside-*O*-acetylpentoside (diosmetin *O*- apiofuranosyl -*O*-acetylapiofuranosyl)	EA, MeOH
53.	C_15_H_10_O_6_	Luteolin	MeOH, INF
54.	C_31_H_28_O_14_	Trihydroxymethoxyflavone *O*-pentoside *O*-hydroxyferuloyl (diosmetin *O*-apiofuranosyl *O*-hydroxyferuloyl)	INF
55.	C_18_H_32_O_5_	Fatty acid	DCM, EA, MeOH, H_2_O, INF
56.	C_11_H_16_O_2_	Unknown	DCM, EA, MeOH
57.	C_18_H_34_O_5_	Fatty acid	DCM, EA, MeOH, H_2_O, INF
58.	C_16_H_12_O_6_	Trihydroxymethoxyflavone (diosmetin)	EA

DCM—dichloromethane; EA—ethyl acetate; MeOH-methanolic; H_2_O—aqueous; INF—infusion.

**Table 2:** Antioxidant properties of the tested extracts *.

Extracts	DPPH (mg TE/g)	ABTS (mg TE/g)	CUPRAC (mg TE/g)	FRAP (mg TE/g)	Phosphomolybdenum Assay (mmol TE/g)	MCA (mg EDTAE/g)
Dichloromethane	5.20 ± 0.33 ^d^	29.53 ± 0.32 ^d^	65.07 ± 1.53 ^c^	17.68 ± 0.54 ^d^	1.23 ± 0.08 ^b^	10.20 ± 0.96 ^c^
Ethyl acetate	10.47 ± 0.91 ^c^	32.65 ± 0.25 ^d^	70.71 ± 0.77 ^c^	19.12 ± 0.15 ^d^	1.66 ± 0.04 ^a^	17.52 ± 0.38 ^b^
Methanol	92.32 ± 0.80 ^a^	227.90 ± 3.36 ^a^	375.36 ± 8.66 ^a^	173.92 ± 3.04 ^a^	1.63 ± 0.15 ^a^	17.38 ± 1.66 ^b^
Water	8.65 ± 0.90 ^c^	65.75 ± 0.89 ^c^	72.70 ± 0.23 ^c^	36.61 ± 1.04 ^c^	0.25 ± 0.01 ^c^	35.91 ± 0.11 ^a^
Infusion	20.75 ± 1.91 ^b^	83.37 ± 1.49 ^b^	108.52 ± 1.74 ^b^	57.24 ± 2.27 ^b^	1.13 ± 0.02 ^b^	na

* Values are reported as mean ± SD of three parallel measurements. MCA: metal chelating activity; TE: trolox equivalent; EDTAE: EDTA equivalent. na: not active. Different letters indicate significant differences in the tested extracts (*p* < 0.05).

**Table 3:** Enzyme inhibitory effects of the tested extracts *.

Extracts	AChE (mg GALAE/g)	BChE (mg GALAE/g)	Tyrosinase (mg KAE/g)	Amylase (mmol ACAE/g)	Glucosidase (mmol ACAE/g)
Dichloromethane	1.51 ± 0.03 ^a^	na	39.24 ± 1.66 ^b^	0.53 ± 0.01 ^b^	2.02 ± 0.10 ^a^
Ethyl acetate	1.63 ± 0.22 ^a^	0.45 ± 0.01 ^b^	24.31 ± 1.60 ^c^	0.58 ± 0.03 ^a^	2.12 ± 0.01 ^a^
Methanol	1.09 ± 0.08 ^b^	1.02 ± 0.09 ^a^	49.83 ± 2.04 ^a^	0.48 ± 0.03 ^b^	0.67 ± 0.02 ^b^
Water	na	na	40.92 ± 2.02 ^b^	0.06 ± 0.01 ^c^	na
Infusion	na	na	38.57 ± 0.99 ^b^	0.09 ± 0.01 ^c^	0.19 ± 0.02 ^c^

* Values are reported as mean ± SD of three parallel measurements. GALAE: galantamine equivalent; KAE: kojic acid equivalent; ACAE: acarbose equivalent; na: not active. Different letters indicate significant differences in the tested extracts (*p* < 0.05).

**Table 4:** Cytotoxicity of *Justicia secunda* extracts after 72 h incubation.

Tested Extracts	VERO	FaDu		Detroit 562	
	CC_50_ *	CC_50_	SI **	CC_50_	SI
Dichloromethane	198.33 ± 11.42	70.18 ± 5.89	2.83	113.53 ± 7.77	1.75
Ethyl acetate	68.00 ± 4.80	61.18 ± 7.83	1.11	79.57 ± 7.34	0.85
Methanol	947.93 ± 40.10	556.90 ± 38.82	1.70	408.33 ± 26.65	2.32
Water	>1000	81.69 ± 5.88	>12.24	127.98 ± 13.75	>7.81
Infusion	>1000	70.67 ± 7.95	>14.15	96.81 ± 13.06	>10.33

* CC_50_ (μg/mL, mean ± SD)—50% cytotoxic concentration; ** SI—anticancer selectivity, tested with reference to VERO cells (CC_50_VERO/CC_50_Cancer), SI > 1 indicates selectivity.

## Data Availability

Not applicable.

## Supplementary Materials

The following supporting information can be downloaded at: https://www.mdpi.com/article/10.3390/antiox12020509/s1. Table S1. Chemical characterization of the tested extracts. The detailed description of Materials and Methods.
