# Supplementary material for: Chemical Characterization of Different Extracts of Justicia secunda Vahl and Determination of Their Anti-Oxidant, Anti-Enzymatic, Anti-Viral, and Cytotoxic Properties
Source: Antioxidants (Basel). 2023 Feb 17;12(2):509. doi: 10.3390/antiox12020509 (PMC9952096; doi:10.3390/antiox12020509)
Supplement: Supplementary file 1 [file antioxidants-12-00509-s001.zip › antioxidants-2211633-supplementary.pdf]

antioxidants

Supplementary Materials

Chemical characterization of different extracts of *Justicia secunda* Vahl and determination of their anti-oxidant, anti-enzymatic, anti-viral, and cytotoxic properties

Łukasz Świątek[1], Elwira Sieniawska[2*], Kouadio Ibrahime Sinan[3], Gokhan Zengin[3], Anastazja Boguszewska[1], Benita Hryć[4], Kouadio Bene[5], Małgorzata Polz-Dacewicz[1], Stefano Dall'Acqua[6]

[1]Department of Virology with SARS Laboratory, Medical University of Lublin, Chodzki 1, 20-093 Lublin, Poland

[2]Department of Natural Products Chemistry, Medical University of Lublin, Chodzki 1, 20-093 Lublin, Poland

[3]Department of Biology, Science Faculty, Selcuk University, Konya, Turkey

[4]MEDICOFARMA Biotech S.A., Zamenhofa 29, 20-453 Lublin, Poland

[5]Laboratoire de Botanique et Phytothérapie, Unité de Formation et de Recherche Sciences de la Nature, Université Nangui Abrogoua, 02 BP 801 Abidjan 02, Abidjan, Cote d'Ivoire

[6]Department of Pharmaceutical and Pharmacological Sciences, University of Padova, Via Marzolo 5, 35131 Padova, Italy

* Correspondence: esieniawska@pharmacognosy.org

Materials and Methods

Assays for Total Phenolic and Flavonoid Contents

The total phenolic content was determined by employing the methods given in the literature with some modification. Sample solution (0.25 mL) was mixed with diluted Folin–Ciocalteu reagent (1 mL, 1:9, v/v) and shaken vigorously. After 3 min, Na_2CO_3 solution (0.75 mL, 1%) was added and the sample absorbance was read at 760 nm after a 2 h incubation at room temperature. The total phenolic content was expressed as milligrams of gallic acid equivalents (mg GAE/g extract) (Uysal et al., 2017).

The total flavonoid content was determined using the $AlCl_3$ method. Briefly, sample solution (1 mL) was mixed with the same volume of aluminum trichloride (2%) in methanol. Similarly, a blank was prepared by adding sample solution (1 mL) to methanol (1 mL) without $AlCl_3$. The sample and blank absorbances were read at 415 nm after a 10 min incubation at room temperature. The absorbance of the blank was subtracted from that of the sample. Rutin was used as a reference standard and the total flavonoid content was expressed as milligrams of rutin equivalents (mg RE/g extract) (Uysal et al., 2017).

Determination of Antioxidant and Enzyme Inhibitory Effects

Antioxidant (DPPH and ABTS radical scavenging, reducing power (CUPRAC and FRAP), phosphomolybdenum and metal chelating (ferrozine method)) and enzyme inhibitory activities (cholinesterase (Elmann's method), tyrosinase (dopachrome method), α-amylase (iodine/potassium iodide method), α -glucosidase (chromogenic PNPG method) and pancreatic lipase (*p*-nitrophenyl butyrate (p-NPB) method) were determined using the methods previously described by Uysal et al. (Uysal et al., 2017) and Grochowski et al. (Grochowski et al., 2017)

For the DPPH (1,1-diphenyl-2-picrylhydrazyl) radical scavenging assay: Sample solution was added to 4 mL of a 0.004% methanol solution of DPPH. The sample absorbance was read at 517 nm after a 30 min incubation at room temperature in the dark. DPPH radical scavenging activity was expressed as milligrams of trolox equivalents (mg TE/g extract).

For ABTS (2,2′-azino-bis(3-ethylbenzothiazoline) 6-sulfonic acid) radical scavenging assay: Briefly, ABTS+ was produced directly by reacting 7 mM ABTS solution with 2.45 mM potassium persulfate and allowing the mixture to stand for 12–16 in the dark at room temperature. Prior to beginning the assay, ABTS solution was diluted with methanol to an absorbance of 0.700 ± 0.02 at 734 nm. Sample solution was added to ABTS solution (2 mL) and mixed. The sample absorbance was read at 734 nm after a 30 min incubation at room temperature. The ABTS radical scavenging activity was expressed as milligrams of trolox equivalents (mg TE/g extract).

For CUPRAC (cupric ion reducing activity) activity assay: Sample solution was added to premixed reaction mixture containing $CuCl_2$ (1 mL, 10 mM), neocuproine (1 mL, 7.5 mM) and NH_4Ac buffer (1 mL, 1 M, pH 7.0). Similarly, a blank was prepared by adding sample solution (0.5 mL) to premixed reaction mixture (3 mL) without $CuCl_2$. Then, the sample and blank absorbances were read at 450 nm after a 30 min incubation at room temperature. The absorbance of the blank was subtracted from that of the sample. CUPRAC activity was expressed as milligrams of trolox equivalents (mg TE/g extract).

For FRAP (ferric reducing antioxidant power) activity assay: Sample solution was added to premixed FRAP reagent (2 mL) containing acetate buffer (0.3 M, pH 3.6), 2,4,6-tris(2-pyridyl)-S-triazine (TPTZ) (10 mM) in 40 mM HCl and ferric chloride (20 mM) in a ratio of 10:1:1 (v/v/v). Then, the sample absorbance was read at 593 nm after a 30 min incubation at room temperature. FRAP activity was expressed as milligrams of trolox equivalents (mg TE/g extract).

For phosphomolybdenum method: Sample solution was combined with 3 mL of reagent solution (0.6 M sulfuric acid, 28 mM sodium phosphate and 4 mM ammonium molybdate). The sample absorbance was read at 695 nm after a 90 min incubation at 95 °C. The total antioxidant capacity was expressed as millimoles of trolox equivalents (mmol TE/g extract).

For metal chelating activity assay: Briefly, sample solution was added to $FeCl_2$ solution (0.05 mL, 2 mM). The reaction was initiated by the addition of 5 mM ferrozine (0.2 mL). Similarly, a blank was prepared by adding sample solution (2 mL) to $FeCl_2$ solution (0.05 mL, 2 mM) and water (0.2 mL) without ferrozine. Then, the sample and blank absorbances were read at 562 nm after 10 min incubation at room temperature. The absorbance of the blank was sub-tracted from that of the

sample. The metal chelating activity was expressed as milligrams of EDTA (disodium edetate) equivalents (mg EDTAE/g extract).

For Cholinesterase (ChE) inhibitory activity assay: Sample solution (was mixed with DTNB (5,5-dithio-bis(2-nitrobenzoic) acid, Sigma, St. Louis, MO, USA) (125 μL) and AChE (acetylcholines-terase (Electric ell acetylcholinesterase, Type-VI-S, EC 3.1.1.7,Sigma)), or BChE (butyrylcholinesterase (horse serum butyrylcholinesterase, EC 3.1.1.8, Sigma)) solution (25 μL) in Tris–HCl buffer (pH 8.0) in a 96-well microplate and incubated for 15 min at 25 °C. The reaction was then initiated with the addition of acetylthiocholine iodide (ATCI, Sigma) or butyrylthiocholine chloride (BTCl, Sigma) (25 μL). Similarly, a blank was prepared by adding sample solution to all reaction reagents without enzyme (AChE or BChE) solution. The sample and blank absorbances were read at 405 nm after 10 min incubation at 25 °C. The absorbance of the blank was subtracted from that of the sample and the cholinesterase inhibitory activity was expressed as galanthamine equivalents (mgGALAE/g extract).

For Tyrosinase inhibitory activity assay: Sample solution was mixed with tyrosinase solution (40 μL, Sigma) and phosphate buffer (100 μL, pH 6.8) in a 96-well microplate and incubated for 15 min at 25 °C. The reaction was then initiated with the addition of L-DOPA (40 μL, Sigma). Similarly, a blank was prepared by adding sample solution to all reaction reagents without enzyme (tyrosinase) solution. The sample and blank absorbances were read at 492 nm after a 10 min incubation at 25 °C. The absorbance of the blank was subtracted from that of the sample and the tyrosinase inhibitory activity was expressed as kojic acid equivalents (mgKAE/g extract).

For α-amylase inhibitory activity assay: Sample solution was mixed with α-amylase solution (ex-porcine pancreas, EC 3.2.1.1, Sigma) (50 μL) in phosphate buffer (pH 6.9 with 6 mM sodium chloride) in a 96-well microplate and incubated for 10 min at 37 °C. After pre-incubation, the reaction was initiated with the addition of starch solution (50 μL, 0.05%). Similarly, a blank was prepared by adding sample solution to all reaction reagents without enzyme (α-amylase) solution. The reaction mixture was incubated 10 min at 37 °C. The reaction was then stopped with the addition of HCl (25 μL, 1 M). This was followed by addition of the iodine-potassium iodide solution (100 μL). The sample and blank absorbances were read at 630 nm. The absorbance of the

blank was subtracted from that of the sample and the α-amylase inhibitory activity was expressed as acarbose equivalents (mmol ACE/g extract).

For α-glucosidase inhibitory activity assay: Sample solution was mixed with glutathione (50 μL), α-glucosidase solution (from Saccharomyces cerevisiae, EC 3.2.1.20, Sigma) (50 μL) in phosphate buffer (pH 6.8) and PNPG (4-N-trophenyl-α-D-glucopyranoside, Sigma) (50 μL) in a 96-well microplate and incubated for 15 min at 37 °C. Similarly, a blank was prepared by adding sample solution to all reaction reagents without enzyme (α-glucosidase) solution. The reaction was then stopped with the addition of sodium carbonate (50 μL, 0.2 M). The sample and blank absorbances were read at 400 nm. The absorbance of the blank was subtracted from that of the sample and the α-glucosidase inhibitory activity was expressed as acarbose equivalents (mmol ACE/g extract).

Cell line maintenance and in vitro experiments

The cytotoxicity of *Justicia secunda* extracts was evaluated *in vitro* towards normal VERO (ECACC, No. 84113001) cells and cancer-derived cell lines – FaDu (ATCC, HTB-43, human hypopharyngeal squamous cell carcinoma), and Detroit 562 (ATCC, No. CCL-138, human pharyngeal cancer), using 3-(4,5-dimethylthiazol-2-yl)-2,5-diphenyltetrazolium bromide (MTT) based protocol.

Media used for *in vitro* culturing included Dulbecco Modified Eagle Medium (DMEM, Corning, Tewksbury, MA, USA) used for VERO cells and Modified Eagle Medium (MEM, Corning) used for pharyngeal carcinomas. Cell media used in the experiments were supplemented with antibiotics (Penicillin-Streptomycin Solution, Corning) and fetal bovine serum (FBS, Corning) – 10% (cell passaging) and 2% (cell maintenance and experiments). Phosphate buffered saline (PBS) and trypsin were bought from Corning, whereas MTT (3-(4,5-dimethylthiazol-2-yl)-2,5-diphenyltetrazolium bromide) and DMSO (dimethyl sulfoxide) from Sigma (Sigma-Aldrich, St. Louis, MO, USA). Incubation was carried out in a 5% CO_2 atmosphere at 37°C (CO_2 incubator, Panasonic Healthcare Co., Tokyo, Japan).

The cytotoxicity testing

Cytotoxicity was tested using an MTT-based protocol following a previously described protocol [Świątek, 2021]. Briefly, the cells were passaged into 96-well plates (Falcon, TC-treated, Corning) and, after overnight incubation, treated with serial dilutions of extract stock solutions for 72 h. Afterwards, the media was removed, cells were washed with PBS, and 10% of MTT solution (5 mg/mL) in cell media was added, and the incubation continued for the next 4 h. Subsequently, the SDS/DMF/PBS (14% SDS, 36% DMF, 50% PBS) solvent was used (100 μL per well) to dissolve the precipitated formazan crystals, and the plates were left at 37°C overnight. Finally, the Synergy H1 Multi-Mode Microplate Reader (BioTek Instruments, Inc. Winooski, Vermont, USA) with Gen5 software (ver. 3.09.07; BioTek Instruments, Inc.) was used to measure the absorbance (540 and 620 nm).

Evaluation of the antiviral properties

The antiviral activity of *Justicia secunda* extracts was tested against HHV-1 (ATCC, Cat. No. VR-260) propagated in the VERO cell line. The antiviral assays involved the influence of extracts on the formation of virus-induced cytopathic effect (CPE), the evaluation of the reduction of infectious titer using the end-point virus titration and the semi-quantitative assessment of the viral load using Real-Time PCR.

Evaluation of the influence on the virus-induced CPE

The infectious titer of HHV-1 used in this study was 5.5±0.25 log$CCID_{50}$/mL ($CCID_{50}$ – 50% cell culture infectious dose). Briefly, the VERO cells (monolayer) in 48-well plates (Falcon, clear flat bottom TC-treated, Corning) were treated (500 μL/well) with HSV-1 suspension (100-fold $CCID_{50}$/mL) in cell media and incubated for 1 hour, leaving at least two uninfected wells as VERO cell control. Afterwards, the media were removed, monolayers washed with PBS, and the non-toxic concentrations of extracts, the highest concentration not exceeding the CC_{10} values, diluted in cell media were added. The non-infected VERO cells (cell control) and non-treated infected cells (virus control) wells were maintained in media containing 2% FBS. The incubation was conducted

until cytopathic effect (CPE) was observed (inverted microscope CKX41, Olympus Corporation, Tokyo, Japan) in virus control, usually approx. 72h. Afterwards, the plates were observed for possible inhibition of CPE by tested extracts compared to the CPE in virus control, and the results were recorded. Lastly, the plates were thrice frozen (-72°C) and thawed; the samples were collected and stored at -72°C until used in the end-point virus titration assay and DNA isolation.

End-point dilution assay for HHV-1 titration

Samples collected from antiviral assays were subjected to an end-point dilution assay to evaluate the HHV-1 titers. Briefly, the VERO cells (monolayer) in 96-well plates were incubated with ten-fold dilutions of samples (3 replicates) in cell media for 72 hours. Daily observation was conducted to monitor the development of CPE. After the incubation, all media were removed, and the virus infectious titer for each sample was measured using the previously described MTT method. Subsequently, the difference (Δlog) of HHV-1 infectious titer ($logCCID_{50}$/mL) in the samples treated with Justicia secunda extracts (JS) and in the virus control (VC) from the same experiment (Δlog = $logCCID_{50}VC$ – $logCCID_{50}JS$) were calculated. The Δlog values were evaluated for every antiviral assay, and the results were expressed further as means of viral titer reduction. A significant antiviral activity can be reported for extracts decreasing the infectious titer by at least 3 log compared to virus control [Świątek, 2021].

Real-Time PCR for HHV-1 viral load

The DNA isolation was carried out using a commercially available kit (QIAamp DNA Mini Kit, Cat#51304, QIAGEN GmbH, Hilden, Germany) following the manufacturer's instructions. The Real-Time PCR amplification was performed using SybrAdvantage qPCR Premix (Takara Bio Inc., Kusatsu, Shiga Prefecture, Japan) and primers (UL54F – 5′ CGCCAAGAAAATTTCATCGAG 3′, UL54R – 5′ ACATCTTGCACCACGCCAG 3′) on the CFX96 thermal cycler (Bio-Rad Laboratories, Inc., California, USA). The amplification cycle parameters were as follows: initial activation (95°C, 20 secs); cycling (40 repeats: denaturation (95°C, 5 secs), annealing and synthesis (60°C, 30 secs), fluorescence acquisition); melting curve analysis (65-95°C). The HHV-1 viral load in the tested

samples was assessed in relation to virus control based on the relative quantity (ΔCq) method using CFX Manager™ Dx Software (Bio-Rad Laboratories).

Table S1. Chemical characterization of the tested extracts (DCM − Dichloromethane; EA − Ethyl acetate; Methanolic; H_2O − Aqueous; INF − Infusion)

Compound no	Retention time	Molecular Formula	NEGATIVE ION MODE			POSITIVE ION MODE			Idenificaction	Extracts	References
			PRECURSOR ION (m/z) measured (Δ, ppm)	(m/z) predicted	Fragment ions (m/z)	PRECURSOR ION (m/z) measured (Δ, ppm)	(m/z) predicted	Fragment ions (m/z)			
1.	1.692	$C_5H_{10}O_6$	[M-H]$^-$ 165.0397 (4.59)	165.0405	165.0372;105.0178; 99.0066; 96.9598; 87.0079; 79.9584; 75.0078; 71.0134	-	-	-	Carboxylic acid derivative	MeOH, H_2O, INF	Fragmentation
2.	1.929	$C_5H_8O_6$	[M-H]$^-$ 163.0239 (5.56)	163.0248	163.0223;145.0119; 117.0174;101.0228; 99.0066; 75.0052; 72.9928; 71.0121	-	-	-	Carboxylic acid derivative	H_2O, INF	Fragmentation
3.	1.932	$C_6H_{10}O_8$	[M-H]$^-$ 209.0294 (4.24)	209.0303	209.0309;191.0172; 133.0109;129.0175; 85.0284; 75.0081; 72.9925; 71.0131	-	-	-	Unknown	MeOH, INF	Fragmentation
4.	2.177	$C_{12}H_{15}NO_6$	[M-H]$^-$ 268.0813 (5.06)	268.0827	250.0743;235.0481; 224.0575;220.0191; 204.0294;165.0093; 138.0201	[M+H]$^+$ 270.0966 (2.28)	270.0972	-	Secundarellone B/C (racemate)	DCM	Theiler et al., 2014, 2017
						[M+Na]$^+$ 292.079 (0.59)	292.0792	246.0707; 274.0688			
5.	2.351	$C_6H_8O_7$	[M-H]$^-$ 191.0179 (9.51)	191.0197	191.0174;158.8439; 129.0173;111.0075;87.0078;85.0282	[M+Na]$^+$ 215.0161 (0.64)	215.0162	-	Citric acid	MeOH, INF	PubChem
6.	2.357	$C_{12}H_{15}NO_5$	[M-H]$^-$ 252.0869 (3.34)	252.0877	237.0601; 222.0440;208.0209; 138.0196; 125.0224;	[2M+Na]$^+$ 529.1798 (-1.05)	529.1793	276.0863; 258.0752	Secundarellone A isomer 1	DCM	Theiler et al., 2014, 2017

						$[M+Na]^+$ 276.0838 (1.75)	276.0842	258.0742			
						$[M+H]^+$ 254.1018 (1.97)	254.1023	236.0942; 208.0962; 176.0688; 170.0774; 166.0475; 155.0557; 134.0587; 116.0488			
7.	6.612	$C_{12}H_{15}NO_5$	$[M-H]^-$ 252.0867 (4.13)	252.0877	237.0584; 222.0460; 138.0177	$[2M+Na]^+$ 529.1790 (0.53)	529.1793	276.0856; 258.0805	Secundarellone A isomer 2	DCM, EA, MeOH, H_2O, INF	Theiler et al., 2014, 2017
						$[M+Na]^+$ 276.0837 (2.15)	276.0842	258.0704			
						$[M+H]^+$ 254.1012 (4.34)	254.1023	236.0922; 208.0950; 176.0701; 170.0813; 166.0474; 155.0603; 134.0598; 116.0512			
8.	7.865	$C_{10}H_{11}NO_6$	$[M-H]^-$ 240.0510 (1.50)	240.0514	222.0367; 178.0476; 166.0473; 151.0364; 138.0166; 123.0049; 116.0321	-	-	-	Unknown	INF	Fragmentation
9.	8.404	$C_{22}H_{12}O_5$	$[M-H]^-$ 355.0638 (-7.31)	355.0612	209.0269; 191.0169; 163.0408; 147.0276; 129.0167; 85.0288;	-	-	-	Lignan derivative	INF	Fragmentation
10.	9.003	$C_{15}H_{20}O_{10}$	$[M-H]^-$ 359.0977 (1.86)	359.0984	197.0423; 182.0183; 153.0516; 138.0286	$[M+Na]^+$ 383.0958 (1.86)	359.0984	221.0320; 185.0465; 181.0473; 174.0479; 139.0692	Syringic acid glucoside	H_2O, INF	PubChem

11.	9.303	$C_7H_{12}O_5$	$[M-H]^-$ 175.0605 (3.96)	175.0612	175.0603; 157.049; 131.0701; 115.0397; 113.0599; 85.0654;	$[M+Na]^+$ 199.0569 (4.51)	199.0577	-	Carboxylic acid derivative	H_2O, INF	Fragmentation
12.	10.262	$C_{13}H_{16}O_8$	$[M-H]^-$ 299.0772 (0.14)	299.0772	255.0876; 138.0252; 137.0225; 93.0337;	$[M+Na]^+$ 323.0735 (0.80)	323.0737	203.0489; 193.0445; 185.0399; 177.0549; 161.0197; 145.0273; 117.0393	Salicylic acid glucoside	MeOH, INF	PubChem Fragmentation
13.	10.741	$C_{13}H_{16}O_9$	$[M-H]^-$ 315.0725 (-1.09)	315.0722	153.0180; 135.0091; 109.0285; 85.0271	-	-	-	Dihydroxybenzoic acid *O*-glucoside (2,4-dihydroxybenzoic acid *O*-glucoside)	INF	Fragmentation
14.	11.221	$C_{12}H_{14}O_8$	$[M-H]^-$ 285.0618 (-0.73)	285.0616	153.0159; 152.0094; 108.0192; 81.0336	-	-	-	Dihydroxybenzoic acid *O*-pentoside	MeOH, H_2O, INF	Fragmentation
15.	11.341	$C_{22}H_{12}O_5$	$[M-H]^-$ 355.0628 (-4.50)	355.0612	209.0260; 191.0162; 147.0261; 129.0179; 85.0276	-	-	-	Lignan derivative (10-(1,3-benzodioxol-5-yl)-5h-benzo[c]furo[3,2-g]chromen-5-one isomer)	INF	Fragmentation
16.	11.885	$C_{13}H_{16}O_9$	$[M-H]^-$ 315.0738 (-5.20)	315.0722	154.0124; 101.0231; 96.9594; 79.9558	-	-	-	Unknown	MeOH, H_2O, INF	Fragmentation
17.	12.300	$C_{22}H_{12}O_5$	$[M-H]^-$ 355.0617 (-1.41)	355.0612	209.0262; 191.0157; 147.0271; 129.0156; 111.0084; 85.0276	-	-	-	Lignan derivative (10-(1,3-benzodioxol-5-yl)-5H-benzo[c]furo[3,2-g]chromen-5-one isomer)	INF	Fragmentation
18.	12.480	$C_{15}H_{18}O_9$	$[M-H]^-$ 341.0849 (8.49)	341.0878	179.0309; 135.0427	-	-	-	Caffeoyl glucoside	MeOH, INF	Fragmentation PubChem

19.	12.899	$C_{18}H_{28}O_9$	$[M-H]^-$ 387.1656 (1.17)	387.1661	163.1143; 119.0334; 113.0289; 101.0234; 89.0229; 71.0132	-	-	-	Hydroxyjasmonic acid glucoside	MeOH, INF	Fragmentation
20.	13.136	$C_{19}H_{30}O_8$	$[M+COOH]^-$ 431.1940 (-4.48)	431.1923	385.1874 ($[M-H]^-$); 223.1326; 161.0431; 153.0914	$[M+Na]^+$ 409.1825 (2.04)	409.1833	248.0673; 203.0502; 185.0387; 177.0793	Roseoside	DCM, EA, MeOH, INF	Silva et al., 2022
21.	13.438	$C_{20}H_{26}O_{12}$	$[M-H]^-$ 457.1330 (4.69)	457.1351	205.0527; 163.0416; 119.0515; 101.0241; 73.0328; 71.0145	$[M+Na]^+$ 481.1297 (4.25)	481.1316	349.0878; 317.0860; 187.0360; 185.0454; 147.0501	Hydroxycinnamic acid *O*-pentoside-glucoside	MeOH, INF	PubChem
22.	14.997	$C_{22}H_{12}O_5$	$[M-H]^-$ 355.0631 (-5.34)	355.0612	209.0275; 191.0167; 163.0338; 147.0276; 129.0165; 111.0065; 85.0283	-	-	-	Lignan derivative (10-(1,3-benzodioxol-5-yl)-5H-benzo[c]furo[3,2-g]chromen-5-one isomer)	INF	Fragmentation
23.	15.236	$C_{17}H_{22}O_{13}$	$[M-H]^-$ 433.0972 (3.60)	433.0988	301.0528; 169.0087; 168.0036; 150.9996; 149.9920; 125.0219; 124.0142	-	-	-	Trihydroxybenzoic acid *O*-dipentoside (Gallic acid *O*-dipentoside)	INF	PubChem Fragmentation
24.	15.476	$C_{12}H_{14}O_9$	$[M-H]^-$ 301.0549 (5.32)	301.0565	169.0071; 168.0056; 151.0006; 149.9959; 125.0220; 124.0163	-	-	-	Trihydroxybenzoic acid *O*-pentoside (Gallic acid *O*-pentoside)	MeOH, INF	PubChem Fragmentation
25.	16.909	$C_{19}H_{32}O_8$	$[M+COOH]^-$ 433.2084 (-1.23)	433.2079	387.2052 ($[M-H]^-$); 316.1571; 119.0445; 102.0273; 101.0238; 89.0229; 71.0110	$[M+Na]^+$ 411.1988 (0.36)	411.1989	203.0491; 189.0954; 177.0538	Cyclohexanone derivative glucoside (Dihydroroseoside)	DCM, EA, MeOH, INF	Fragmentation
26.	18.538	$C_{14}H_{14}O_8$	$[M-H]^-$ 309.0611 (1.58)	309.0616	179.0311; 161.0223; 145.0115; 135.0435	-	-	-	Caffeic acid derivative	MeOH	Fragmentation
27.	20.091	$C_9H_8O_3$	$[M-H]^-$ 163.0406 (-3.24)	163.0401	119.0513; 117.0352; 93.0355; 91.0568	-	-	-	Hydroxycinnamic acid	INF	PubChem Fragmentation

28.	20.337	$C_{11}H_{16}O_3$	-	-	-	[M+H]+ 197.1170 (1.13)	197.1172	197.1187; 179.1075; 161.0961; 135.1173; 133.1019; 107.0867; 105.0695; 91.0541	Unknown	DCM, EA, MeOH, INF	Fragmentation
						[M+Na]+ 219.0984 (3.9)	219.0992	165.0694; 116.0479; 89.0389			
29.	21.534	$C_{33}H_{40}O_{20}$	[M-H]- 755.2011 (3.86)	755.2040	593.1425; 285.0338	[M+H]+ 757.2180 (0.75)	757.2186	611.1582; 449.1018; 433.1066; 287.0524	Luteolin 7-*O*-[β-glucopyranosyl-(1→2)-β-rhamnosyl-(1→6)] β-glucopyranoside	EA, MeOH, INF	Koffi et al., 2013
30.	22.967	$C_{33}H_{40}O_{19}$	[M-H]- 739.2066 (3.38)	739.2091	593.1550; 561.1121; 329.0920; 285.0331; 269.0419	[M+H]+ 741.2235 (0.21)	741.2237	595.1629; 433.1081; 287.0522; 271.0574; 147.0603; 85.0304	Trihydroxyflavone di-*O*-hexoside-*O*-rhamnoside (apigenin 7-*O*-glucoside-glucoside-rhamnoside)	MeOH, INF	Fragmentation
31.	23.202	$C_{21}H_{36}O_8$	[M+COOH]- 461.2383 (2.21)	461.2392	415.2334 ([M-H]-); 387.0261; 174.9569; 119.0341; 101.0204; 89.0244; 71.0157	[M+Na]+ 439.2293 (2.26)	439.2302	421.2243; 369.1954; 118.0820	Decalin derivative	DCM, EA, MeOH, INF	Fragmentation
32.	23.207	$C_{34}H_{42}O_{20}$	[M-H]- 769.2216 (-2.51)	769.2197	299.0541; 284.0279	[M+H]+ 771.2346 (-0.49)	771.2342	-	Trihydroxymethoxyflavone di-*O*-hexoside-*O*-rhamnoside (diosmetin 7-*O*-glucoside-rhamnoside-glucoside)	MeOH, INF	Fragmentation
33.	23.327	$C_{27}H_{30}O_{15}$	[M-H]- 593.1540 (-4.72)	593.1512	285.0356	[M+H]+ 595.1649 (1.43)	595.1657	449.1044; 287.0512	Tetrahydroxyflavone *O*-hexoside-*O*-rhamnoside (luteolin-7-*O*-rutinoside)	EA, MeOH, INF	KOFFI et al., 2020
34.	23.392	$C_{27}H_{30}O_{16}$	[M-H]- 609.1490 (-4.74)	609.1461	300.0275; 301.0338; 271.0263; 179.0003; 151.0020	-	-	-	Rutin	MeOH	PubChem

35.	23.742	$C_{19}H_{32}O_7$	$[M+COOH]^-$ 417.2129 (0.29)	417.2130	371.2052 ($[M-H]^-$); 101.0215; 71.0125	$[M+Na]^+$ 395.2026 (3.83)	395.2040	305.1568; 233.1640; 182.1845; 118.0796	Cyclohexanone derivative glucoside (9-Hydroxy-7-megastigmen-3-one glucoside)	DCM, EA, MeOH, INF	Fragmentation
36.	24.111	$C_{26}H_{34}O_{10}$	$[M-H]^-$ 505.2076 (0.63)	505.2079	265.0505; 137.0256; 93.0353	-	-	-	Lignan derivative	MeOH	Fragmentation; Corrêa & Alcântara, 2012
37.	24.161	$C_{19}H_{28}O_7$	$[M+COOH]^-$ 413.1832 (-4.06)	413.1817	367.1755 ($[M-H]^-$); 327.8006; 89.0216; 81.0287; 73.0246	$[M+H]^+$ 369.1901 (1.85)	369.1908	207.1387; 189.1244; 161.1325; 119.0856; 105.0706	Cyclohexanone derivative	DCM, EA, MeOH	Fragmentation
						$[M+Na]^+$ 391.173 (-0.75)	391.1727	218.1428; 189.4323; 175.1096; 177.0396			
38.	24.586	$C_{37}H_{38}O_{19}$	$[M-H]^-$ 785.1898 (4.65)	785.1935	649.1328; 593.1463; 285.0311; 233.0256; 191.0156; 135.0216; 72.9927	$[M+H]^+$ 787.2051 (3.70)	787.2080	641.1448; 449.1058; 287.0611; 175.0238; 127.0404; 71.0524	Tetrahydroxyflavone *O*-hexoside-*O*-rhamnoside derivative (luteolin *O*-hydroxyferuloyl -*O*-rutinoside)	INF	Fragmentation
39.	25.126	$C_{21}H_{38}O_8$	$[M+COOH]^-$ 463.2536 (3.04)	463.2549	417.2490 ($[M-H]^-$); 280.0896; 182.0400; 161.0467; 89.0269	$[M+Na]^+$ 441.2449 (2.37)	441.2459	263.1970; 203.0504	Decalin glucoside derivative (Ophiopogonoside A isomer)	EA, MeOH, INF	Fragmentation
40.	25.370	$C_{26}H_{28}O_{15}$	$[M-H]^-$ 579.1353 (0.42)	579.1355	417.0821; 399.0743; 285.0399	$[M+H]^+$ 581.1494 (1.20)	581.1501	419.0937; 287.0529	Tetrahydroxyflavone *O*-pentoside-hexoside; (graveobioside A = luteolin 7-*O*-(2-apiosyl)glucoside)	EA, MeOH, INF	Fragmentation
41.	26.019	$C_{13}H_{15}NO_3$	$[M-H]^-$ 232.0985 (-2.5)	232.0979	217.0733; 188.0356; 160.0403; 132.0476	$[M+H]^+$ 234.1133 (-3.56)	234.1125	206.1150; 195.9082; 176.0706174.0909; 146.0957; 117.0605; 91.0520	Unknown	DCM	Fragmentation

						[M+Na]+ 256.0931 (5.64)	256.0944	239.1218; 215.9169; 187.1256; 183.1135; 135.1169; 122.0972; 84.9538			
42.	26.444	$C_{25}H_{26}O_{14}$	[M-H]- 549.1223 (4.87)	549.1250	417.0759; 327.0450; 285.0363	[M+H]+ 551.1414 (-3.40)	551.1395	419.0976; 287.0548	Luteolin 7-*O*-[β-apiofuranosyl-(1→2)]-β-xylopyranoside	EA, MeOH, INF	Koffi et al., 2013
43.	26.499	$C_{19}H_{34}O_{10}$	[M+COOH]- 467.2148 (-3.32)	467.2134	421.2070 ([M-H]-); 289.1654; 161.0433; 113.0234; 101.0222; 85.0284; 71.0145	[M+Na]+ 445.2044 (0.04)	445.2044	333.0807; 137.0718; 105.0680	Cyclohexanone derivative glucoside	DCM, EA, MeOH, INF	Fragmentation
44.	26.803	$C_{26}H_{28}O_{14}$	[M-H]- 563.1407 (-0.13)	563.1406	417.0812; 285.0373	[M+H]+ 565.1564 (-2.16)	565.1552	419.0955; 287.0566	Tetrahydroxyflavone *O*-pentoside-*O*-rhamnoside (luteolin *O*-pentoside-*O*-rhamnoside)	EA, MeOH, INF	Fragmentation
45.	27.098	$C_{17}H_{30}O_{7}$	[M+COOH]- 391.1984 (-3.02)	391.1974	345.1893 ([M-H]-); 278.9318; 161.0452; 101.0271; 89.0231; 85.0277; 71.0119	[M+H]+ 369.1877 (1.95)	369.1884	201.0293; 175.0539;77.0391	Unknown	DCM, EA, MeOH, INF	Fragmentation
46.	27.517	$C_{21}H_{38}O_{8}$	[M+COOH]- 463.2552 (-0.79)	463.2549	417.2479 ([M-H]-); 161.0444; 113.0221; 119.0486; 101.0227; 71.0140	[M+Na]+ 441.2432 (6.43)	441.2459	295.0154; 280.216; 163.0611; 109.0350; 85.0328; 73.0276	Decalin glucoside derivative (Ophiopogonoside A isomer)	DCM, EA, MeOH, INF	Fragmentation
47.	27.640	$C_{19}H_{36}O_{10}$	[M+COOH]- 469.2285 (1.3)	469.2291	423.2212 ([M-H]-); 291.1795; 161.0438; 113.0230; 101.0232; 71.0147	[M+Na]+ 447.2195 (1.34)	447.2201	315.1726; 275.0702; 167.1365; 136.0664	Rhodiooctanoside	DCM, EA, MeOH,	Fragmentation

48.	28.007	$C_{27}H_{28}O_{15}$	$[M-H]^-$ 591.1363 (-1.28)	591.1355	549.1283; 531.1164; 285.0412	$[M+H]^+$ 593.1533 (-5.41)	591.1355	419.1091; 287.0566; 175.0597	Tetrahydroxyflavone *O*-pentoside-*O*-acetylpentoside (luteolin *O*-apiofuranosyl -*O*-acetylapiofuranosyl)	MeOH	Fragmentation
49.	28.301	$C_{25}H_{26}O_{13}$	$[M-H]^-$ 533.1277 (4.43)	533.1301	269.0473; 181.0546	$[M+H]^+$ 535.1466 (-3.71)	535.1446	403.1044; 271.0595; 153.0127; 133.0455	Trihydroxyflavone di-*O*-pentoside (apigenin di-*O*-pentoside)	MeOH, INF	Fragmentation
50.	28.481	$C_{26}H_{28}O_{14}$	$[M-H]^-$ 563.1397 (1.65)	563.1406	431.0857; 299.0514; 284.0289	$[M+H]^+$ 565.1569 (-3.05)	565.1552	433.1121; 301.0701; 286.0508	Trihydroxymethoxyflavone di-*O*-pentoside (diosmetin 7-*O*-apiofuranosyl-xylopyranoside)	EA, MeOH, INF	Fragmentation
51.	28.901	$C_{27}H_{30}O_{14}$	$[M-H]^-$ 577.1524 (6.71)	577.1563	445.0708; 299.0533; 284.0277; 255.0273; 232.0934	$[M+H]^+$ 579.1715 (-1.16)	579.1708	433.1186; 301.0749; 286.0437	Trihydroxymethoxyflavone *O*-pentoside *O*-rhamnoside (diosmetin *O*-apiofuranosyl- *O*-rhamnoside)	EA, MeOH, H_2O, INF	Fragmentation
52.	30.757	$C_{28}H_{30}O_{15}$	$[M-H]^-$ 605.1506 (0.98)	605.1512	563.1392; 545.1310; 530.1057; 299.0541; 284.0309; 255.0278	$[M+H]^+$ 607.1639 (3.05)	607.1657	433.1083; 301.0715; 286.0485; 97.0312	Trihydroxymethoxyflavone *O*-pentoside-*O*-acetylpentoside (diosmetin *O*-apiofuranosyl -*O*-acetylapiofuranosyl)	EA, MeOH	Fragmentation
						$[M+Na]^+$ 629.1460 (2.79)	629.1477	-			
53.	31.238	$C_{15}H_{10}O_6$	$[M-H]^-$ 285.0405 (-0.13)	285.0405	175.0377; 151.0046; 149.0251; 133.0293; 107.0120	$[M+H]^+$ 287.0555 (-1.70)	287.0550	-	Luteolin	MeOH, INF	Fragmentation PubChem

54.	32.137	$C_{31}H_{28}O_{14}$	$[M-H]^-$ 623.1361 (7.26)	623.1406	431.1076; 323.0640; 299.0628; 233.0346; 135.0361; 191.0197; 72.9946	$[M+H]^+$ 625.1434 (-5.57)	625.1399	-	Trihydroxymethoxyflavone *O*-pentoside *O*-hydroxyferuloyl (diosmetin *O*-apiofuranosyl *O*-hydroxyferuloyl)	INF	Fragmentation
55.	32.317	$C_{18}H_{32}O_5$	$[M-H]^-$ 327.2175 (0.60)	327.2177	229.1431; 211.1366; 183.1417; 171.1041; 97.0630; 85.0299	$[M+Na]^+$ 351.2158 (-4.89)	351.2142	351.2185; 236.1367; 195.1064; 179.0860; 166.0818; 83.0979	Fatty acid	DCM, EA, MeOH, H_2O, INF	Fragmentation
56.	32.742	$C_{11}H_{16}O_2$	-	-	-	$[M+H]^+$ 181.1217 (3.37)	181.1223	163.1106; 135.1161; 107.0843; 91.0547; 93.0708; 79.0547; 77.0389	Unknown	DCM, EA, MeOH	Fragmentation
						$[M+Na]^+$ 203.1035 (4.17)	203.1043	147.0429; 131.0840; 129.0665; 93.0585; 91.0610			
57.	33.993	$C_{18}H_{34}O_5$	$[M-H]^-$ 329.2340 (-1.98)	329.2333	229.1439; 211.1356; 183.1394; 171.1033; 139.1133; 127.1121; 99.0812	$[M+Na]^+$ 353.2297 (0.44)	353.2298	353.2252; 271.1220; 190.0905; 160.0732; 120.0815; 118.0648; 91.0544	Fatty acid	DCM, EA, MeOH, H_2O, INF	Fragmentation
58.	34.414	$C_{16}H_{12}O_6$	$[M-H]^-$ 299.0565 (-1.29)	299.0561	284.0325; 256.0371; 228.0417; 227.0306; 151.0019; 132.0170; 107.0133	$[M+H]^+$ 301.0699 (2.55)	301.0707	-	Trihydroxymethoxyflavone (diosmetin)	EA	Fragmentation PubChem

Supplementary material references

Grochowski, D. M., Uysal, S., Aktumsek, A., Granica, S., Zengin, G., Ceylan, R., Locatelli, M., & Tomczyk, M. (2017). In vitro enzyme inhibitory properties, antioxidant activities, and phytochemical profile of Potentilla thuringiaca. Phytochemistry Letters, 20, 365-372. https://doi.org/10.1016/j.phytol.2017.03.005

Świątek, Ł.; Sieniawska, E.; Sinan, K.I.; Maciejewska-Turska, M.; Boguszewska, A.; Polz-Dacewicz, M.; Senkardes, I.; Guler, G.O.; Bibi Sadeer, N.; Mahomoodally, M.F.; Zengin, G. LC-ESI-QTOF-MS/MS Analysis, Cytotoxic, Antiviral, Antioxidant, and Enzyme Inhibitory Properties of Four Extracts of *Geranium pyrenaicum* Burm. f.: A Good Gift from the Natural Treasure. *Int. J. Mol. Sci.* 2021, *22*, 7621. https://doi.org/10.3390/ijms22147621

Uysal, S., Zengin, G., Locatelli, M., Bahadori, M. B., Mocan, A., Bellagamba, G., De Luca, E., Mollica, A., & Aktumsek, A. (2017). Cytotoxic and Enzyme Inhibitory Potential of Two Potentilla species (P. speciosa L. and P. reptans Willd.) and Their Chemical Composition. Frontiers in Pharmacology, 8. https://doi.org/10.3389/fphar.2017.00290

Theiler, B. A.; Revoltella, S.; Zehl, M.; Dangl, C.; Caisa, L. O. E.; König, J.; Winkler, J.; Urban, E.; Glasl, S., Secundarellone A, B, and C from the leaves of Justicia secunda Vahl. Phytochemistry Letters 2014, 10, cxxix-cxxxii.

e Silva, J. P.; Pereira, L. C.; Abreu, L. S.; Lins, F. S.; de Souza, T. A.; do Espírito-Santo, R. F.; Barros, R. P.; Villarreal, C. F.; de Melo, J. I.; Scotti, M. T., Targeted Isolation of Anti-inflammatory Lignans from Justicia aequilabris by Molecular Networking Approach. Journal of Natural Products 2022, 85, (9), 2184-2191.

Koffi, E. N.; Le Guernevé, C.; Lozano, P. R.; Meudec, E.; Adjé, F. A.; Bekro, Y.-A.; Lozano, Y. F., Polyphenol extraction and characterization of Justicia secunda Vahl leaves for traditional medicinal uses. Industrial Crops and Products 2013, 49, 682-689.

Koffi, E.; Kassi, A. B. B.; Adje, F. A.; Lozano, Y. F.; Bekro, Y.-A., Effect of freeze-drying and spray-drying on total phenolics content and antioxidant activity from aqueous extract of Justicia secunda leaves. Trends in Phytochemical Research 2020, 4, (2), 69-76.

Corrêa, G. M.; Alcântara, A. F. d. C., Chemical constituents and biological activities of species of Justicia: a review. Revista Brasileira de farmacognosia 2012, 22, 220-238.
